# A unique subseafloor microbiosphere in the Mariana Trench driven by episodic sedimentation

**DOI:** 10.1007/s42995-023-00212-y

**Published:** 2024-01-23

**Authors:** Jiwen Liu, Da-Wei Li, Xinxin He, Ronghua Liu, Haojin Cheng, Chenglong Su, Mengna Chen, Yonghong Wang, Zhongsheng Zhao, Hanyue Xu, Zhangyu Cheng, Zicheng Wang, Nikolai Pedentchouk, David J. Lea-Smith, Jonathan D. Todd, Xiaoshou Liu, Meixun Zhao, Xiao-Hua Zhang

**Affiliations:** 1 Frontiers Science Center for Deep Ocean Multispheres and Earth System, and College of Marine Life Sciences, Ocean University of China, Qingdao, 266003 China; 2 Laboratory for Marine Ecology and Environmental Science, Laoshan Laboratory, Qingdao, 266237 China; 3 Institute of Evolution and Marine Biodiversity, Ocean University of China, Qingdao, 266003 China; 4 Key Laboratory of Marine Chemistry Theory and Technology, Ministry of Education, Ocean University of China, Qingdao, 266100 China; 5 Key Lab of Submarine Geosciences and Prospecting Techniques, Ministry of Education/College of Marine Geosciences, Ocean University of China, Qingdao, 266100 China; 6 Key Laboratory of Physical Oceanography, Ministry of Education/Research Vessel Centre, Ocean University of China, Qingdao, 266100 China; 7 School of Environmental Sciences, University of East Anglia, Norwich, NR4 7TJ UK; 8 School of Biological Sciences, University of East Anglia, Norwich, NR4 7TJ UK

**Keywords:** Hadal subseafloor, Deep water sediment, Mariana Trench, Radiocarbon, Microbial community, Redox potential

## Abstract

Hadal trenches are characterized by enhanced and infrequent high-rate episodic sedimentation events that likely introduce not only labile organic carbon and key nutrients but also new microbes that significantly alter the subseafloor microbiosphere. Currently, the role of high-rate episodic sedimentation in controlling the composition of the hadal subseafloor microbiosphere is unknown. Here, analyses of carbon isotope composition in a ~ 750 cm long sediment core from the Challenger Deep revealed noncontinuous deposition, with anomalous ^14^C ages likely caused by seismically driven mass transport and the funneling effect of trench geomorphology. Microbial community composition and diverse enzyme activities in the upper ~ 27 cm differed from those at lower depths, probably due to sudden sediment deposition and differences in redox condition and organic matter availability. At lower depths, microbial population numbers, and composition remained relatively constant, except at some discrete depths with altered enzyme activity and microbial phyla abundance, possibly due to additional sudden sedimentation events of different magnitude. Evidence is provided of a unique role for high-rate episodic sedimentation events in controlling the subsurface microbiosphere in Earth’s deepest ocean floor and highlight the need to perform thorough analysis over a large depth range to characterize hadal benthic populations. Such depositional processes are likely crucial in shaping deep-water geochemical environments and thereby the deep subseafloor biosphere.

**Supplementary Information:**

The online version contains supplementary material available at 10.1007/s42995-023-00212-y.

## Introduction

Subseafloor environments, generally > 1.5 m below the seafloor (D'Hondt et al. 2009), are characterized by low bioavailable organic carbon (OC) content due to a significant loss of labile organic compounds during deposition and early stages of diagenesis (Jørgensen and Marshall. 2016; Zhao et al. 2020). Therefore, microbial life inhabiting the subseafloor experiences extreme energy limitation, resulting in lower cell abundance and metabolic activity (Kallmeyer et al. 2012; Orsi et al. 2020; Parkes et al. 2014). Despite these limitations, deep seabed microbial populations likely exceed the abundance of those in the overlying water column (Jørgensen and Marshall. 2016; Kallmeyer et al. 2012; Parkes et al. 2014).

Accumulation of OC in sediments generally declines with increasing water depth (Estes et al. 2019; Faust et al. 2021; Hayes et al. 2017). However, the hadal zone, with water depths 6000–11,000 m below sea level (mbsl) and almost exclusively comprised of trenches, does not follow this trend (Danovaro et al. 2003; Glud et al. 2013; Hiraoka et al. 2020; Luo et al. 2017) because of enhanced sediment deposition compared to the surrounding abyssal plains (Glud et al. 2013; Luo et al. 2018; Wenzhöfer et al. 2016). This has been attributed to seismically driven mass-wasting events (e.g., slumping, debris flows) along the slopes (Bao et al. 2018; Heeszel et al. 2008; Kioka et al. 2019) and the funneling effect facilitated by the V-shaped geomorphology of the trenches (Luo et al. 2017; Turnewitsch et al. 2014).

Hadal trenches are considered as depocenters of OC (Danovaro et al. 2003). Consequently, higher microbial populations and OC consumption have been reported in hadal trench sediments compared with adjacent abyssal sites (Glud et al. 2013; Luo et al. 2018; Wenzhöfer et al. 2016). This enhanced hadal benthic OC consumption is reflected in the higher reported abundance of prokaryotic cells and viruses in hadal compared to abyssal sediments, with the difference being more evident in subsurface sediments below 10 cm than in the surface layer (Glud et al. 2013; Schauberger et al. 2021a). The extent of microbial activity and organic content are likely controlled by the sediment deposition rate, which remains largely unknown due to a lack of well-preserved planktonic foraminiferal shell material for radiocarbon (^14^C) dating (Feely et al. 2004). Additionally, the extent to which the hadal subseafloor microbes are affected by enhanced and/or sudden OC deposition is currently unknown, though, it is perhaps an important factor in understanding the role of hadal trenches in the global ocean carbon cycle.

Microbial communities in hadal sediments typically show a large compositional variation between the trench slope and bottom, which has been attributed to differences in water pressure, OC content and the rates of sedimentation (Fan et al. 2022; Hiraoka et al. 2020; Zhou et al. 2022; Zhu et al. 2023). There are also intra- and inter-trench community differences shaped mainly by depositional dynamics along the trench axis or between trenches (Hiraoka et al. 2020; Liu et al. 2019; Peoples et al. 2019) (Supplementary Table S1). In these and other trench studies (Chen et al. 2021; Schauberger et al. 2021b; Thamdrup et al. 2021), analysis was primarily limited to the upper sediment layers (0–30 cm below seafloor (cmbsf)), which contain large amounts of recently deposited material and associated microbial populations. The studies that examined deeper sediment to a maximum of 155 cmbsf did not perform metagenomic analysis and only investigated a few discrete depths (Hiraoka et al. 2020; Li et al. 2019; Nunoura et al. 2018; Rastelli et al. 2019) (Supplementary Table S1), which made it challenging to determine the metabolic potential and distribution of microbial populations in the deeper sediments.

**Fig. 1:**
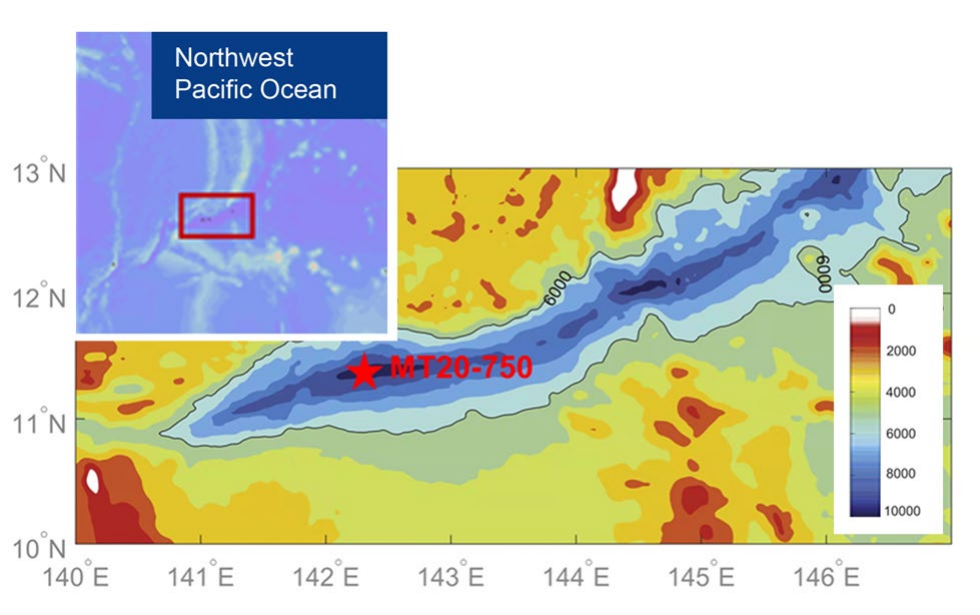
Map showing ocean bathymetry of the southern Mariana Trench and the location of the core site MT20-750 (red star). The bathymetric data were sourced from the ETOPO1 (https://www.ngdc.noaa.gov/mgg/global/relief/ETOPO1/)

To address these knowledge gaps, geochemical and microbial community analyses were performed on a sediment core of 752 cm in length, collected from the Mariana Trench, Challenger Deep (Fig. 1). Our aim is to determine how the microbiosphere in the hadal subseafloor responds to physicochemical environmental changes caused by episodic sedimentation events.

## Materials and methods

### Sampling

A 752 cm long sediment core was collected by the research vessel *Dongfanghong* 3 in July 2020 from the Challenger Deep in the Mariana Trench (MT20-750, 11° 19.904ʹ N, 142° 12.083ʹ E) using a gravity corer (Cheng et al. 2023) (Fig. 1). The sampling site had a water depth of 10,816 m. The core was stored at – 80 °C onboard and was then processed when it was brought back to the laboratory. The core was sliced and subsampled using clean autoclaved sterile spatulas. To avoid contamination, the outer 1 cm of sediment was removed and the uncontaminated center of the core sample was sampled. Sediment samples were taken as 1–3 cm thick slices in the upper 178 cm and to avoid contamination from adjacent sediment layers, the inner depth of each slice was used, most notably for microbial analysis. Below this, sampling was performed at 25–50 cm intervals. A total of 81 subsamples were obtained for microbial community analysis and these were transferred to sterilized plastic tubes and stored at – 80 °C. Sediment porewater was extracted by centrifugation at 2600×g for 10 min (Hiraoka et al. 2020) and the seawater supernatant was filtered using a 0.2-µm syringe cartridge before storage at – 80 °C. Samples for total organic carbon (TOC) and total nitrogen (TN) measurements were stored at – 20 °C. Porewater nutrients (NO_3_^−^, NO_2_^−^, NH_4_^+^, PO_4_^3−^, SiO_3_^2−^ and SO_4_^2−^), sediment TOC, TOC-δ^13^C, TN and sediment particle sizes were measured at 37 discrete depths spanning the whole core (Supplementary Table S2). TOC-^14^C was measured at 25 depths from 0 to 490 cm (Supplementary Table S2).

### Geochemical analyses

The porewater nutrients were measured using a QuAAtro continuous-flow analyzer (SEAL, Germany). Porewater sulfate was analyzed using ion chromatography (CIC-D100, Shine, China) on 1:80 diluted aliquots in Milli-Q water. TOC and TN were analyzed using an elemental analyzer FLASH 2000 following the method of Chen et al. (2023). Analysis standard deviation (precision) for TOC and TN were ± 0.02 wt% (*n* = 6) and ± 0.01 wt% (*n* = 6), respectively, determined by replicate analysis of atropine (Thermo Fisher Scientific, Netherlands) and a low organic content soil (Elemental Microanalysis Ltd., UK). Another aliquot of samples was oven-dried and placed in a ceramic boat for analysis of TOC-δ^13^C using a FLASH elemental analyzer 1112 series coupled with a Thermo Fisher Delta V isotopic ratio mass spectrometer and reported in per mil (× 10^–3^) using δ notation relative to the V-PDB standard (Chen et al. 2023). Sediment particle sizes were measured with a Mastersize 3000 Laser Analyzer.

To provide information on the sedimentary depositional process, the TOC-^14^C profile was further analyzed using a mini carbon dating system (MICADAS, Ionplus AG, Switzerland) at the Ocean University of China radiocarbon Accelerator Mass Spectrometry center (OUC-CAMS), following the method of Chen et al*.* (2023). The radiocarbon content of TOC was reported as fraction modern (*Fm*) corrected for carbon isotopic fractionation that occurred during sample formation and processing. Radiocarbon age was reported as absolute radiocarbon age (relative to 1950 BP) using equation: ^14^C age = 8267 × ln (*Fm*). Both changes in the proportion of terrestrial versus marine sources and the marine reservoir effect contribute to the ^14^C age of sediment OC (Chen et al. 2023).

### Cell counting

Counting of microbial cells was performed via flow cytometry according to the method previously described (Frossard et al. 2016; Khalili et al. 2019) with certain modifications. Briefly, 0.5 mL (~ 0.7 g) of each subsample was fixed with 4 mL of 0.22 μm-filtered (Millex^®^-GP, Millipore, Wohlen, Switzerland) sterile seawater containing 1% paraformaldehyde (PFA). Fixed samples were stored at 4 °C for 1 h before processing. To extract microbial cells from sediment, the fixed slurry samples were washed with 0.5 mL of detergent solution (10 mmol/L tetrasodium pyrophosphate with pH 8.0 containing Tween 80 at 0.1% final concentration). The solution was subjected to vortex mixing for 30 s, stored at 4 °C for 30 min, and then subjected to low-power ultrasonic treatment for 90 s (JSP Ultrasonic Cleaners, model US21l; 50 W, 50 Hz). Cells and abiotic particles were separated by centrifugation at 719 × g for 15 min. The supernatant containing washed cells was collected and mixed with SYBR Green I (v/v, 75:1). The mixture was incubated in the dark for 15 min and analyzed with a BD FACSJazzTM flow cytometer (Becton Dickinson) equipped with a 200-mW solid-state laser emitting light at 488 nm. Three parallel measures were performed for each sample. For each measurement, cells were extracted three times, and the sum of three times was used as the cell abundance.

### Extracellular enzyme activity

Fluorogenic substrates were used to measure the potential enzymatic activity of extracellular hydrolytic enzymes (Liang et al. 2021; Mahmoudi et al. 2020). A 20 mmol/L stock solution of each fluorogenic substrate (Supplementary Table S3) dissolved in DMSO was stored in the dark at – 20 °C. Sediments from each depth were mixed with reaction buffers (0.2 mol/L borate-buffered saline solution with pH 7.4 for N-acetyl-*β*-D-glucosaminidase and aminopeptidase, pH 8.0 for *β*-D-cellobiohydrolase and pH 7.8 for others; 0.1 mol/L pH 6.0 MES buffer for lipase; the pH value for each enzyme was determined for minimum substrate spontaneous degradation) in a vortex (0.05 g/mL). Triplicate samples containing 90 μL of slurry were dispensed into 96-well Costar^®^ assay plates (Corning, USA) and then amended with 10 μL of fluorogenic substrates to a final concentration of 200 μmol/L. Autoclaved sterile and buffer only controls were also included. Fluorescence was measured using a microplate fluorometer and luminometer (Thermo Fisher Scientific, Fluoroskan Ascent FL; excitation = 365 nm, emission = 440 nm). The enzymatic activity measurements and fluorescent signal detection were performed at room temperature. The high temperature may accelerate the reaction rate facilitating comparison across depths, although it may not reflect the in situ enzyme activity. Fluorescent signal released from degraded substrates was read every 1 min over a period of 3 h. Hydrolysis rates were calculated using the fluorescence values that changed linearly with time and were normalized to fluorescence signals released per gram of wet sediment per minute.

### DNA extraction, 16S rRNA gene high-throughput sequencing and quantitative PCR

Genomic DNA was extracted from 0.25 g of wet weight sediment using the MO BIO PowerSoil DNA Isolation Kit. To increase the efficiency of cell disruption, a FastPrep-24 homogenizer (MP Biomedicals) was utilized. Quality (contamination by other absorbing compounds) and quantity of the extracted DNA were measured using a Nanodrop spectrophotometer ND-2000 (Thermo Fisher Scientific). The universal primer set 515FmodF (5′-GTGYCAGCMGCCGCGGTAA-3′) and 806RmodR (5′-GGACTACNVGGGTWTCTAAT-3′) were used for 16S rRNA gene amplification targeting the V4 hypervariable region (Walters et al. 2016). Gene amplification was carried out under the following parameters: 95 °C for 3 min, 29 cycles of 95 °C for 30 s, 55 °C for 30 s and 72 °C for 45 s, followed by a final extension for 10 min at 72 °C. The reactions were run in triplicate using a 20 μL mixture containing 0.4 μL of FastPfu polymerase, 0.8 μL of each primer (5 μmol/L), 2 μL of 2.5 mmol/L deoxyribonucleoside triphosphates (dNTPs), 4 μL of 5 × FastPfu Buffer, and 10 ng of template DNA. Purification of the amplified products was performed using the AxyPrep DNA Gel Extraction Kit (Axygen Biosciences, Union City, CA, USA). Purified amplicons were pooled in equimolar and paired-end sequenced (2 × 300) on an Illumina Miseq PE300 platform (Illumina, San Diego, USA) at Majorbio Bio-Pharm Technology Co. Ltd. (Majorbio, Shanghai, China).

The abundance of total bacteria and archaea was measured by 16S rRNA gene based quantitative PCR (qPCR) with the primer set 967R/1046R (Sogin et al. 2006) and 967F/1060R (Cadillo-Quiroz et al. 2006), respectively. These primer sets may introduce amplification bias but can provide sufficient information on the spatiotemporal shifts of microbial abundance (Liu et al. 2022; Sogin et al. 2006). A 20 µL reaction system contained 10 µL of SYBR Premix ExTaq II (2×), 0.4 µL of ROX (50×) (TaKaRa, Tokyo, Japan), 0.4 µL (0.8 µL for archaea; 10 µmol/L) of primers and 2 µL of template. The thermal cycling parameters consisted of an initial denaturation step at 95 °C for 5 min, 40 cycles of 95 °C for 30 s, 53 °C for 1 min and 72 °C for 15 s, and a final extension at 72 °C for 10 min for bacterial 16S rRNA gene. For archaeal 16S rRNA gene, the thermal cycling parameters consisted of an initial denaturation step at 95 °C for 30 s, 40 cycles of 95 °C for 5 s, 50 °C for 30 s and 72 °C for 30 s, and a final extension at 72 °C for 5 min. Runs for each sample with negative controls were conducted in triplicate, using a QuantStudio^™^ 5 System (Thermo Fisher Scientific). Standard curves were generated by PCR amplification of a tenfold serial dilution of plasmids containing target gene fragments. The amplification curves exhibited clear linear relationships (*R*^2^ > 0.999) and yielded an amplification efficiency of 0.90 and 0.95 for bacterial and archaeal 16S rRNA gene, respectively.

### Read processing and statistical analysis

Raw reads were quality-filtered by Trimmomatic (Bolger et al. 2014) with the following criteria: (1) reads were truncated at any site receiving an average quality score < 20 over a 50 bp sliding window; (2) reads longer than 100 bp in length, having no ambiguous bases, no mismatch to barcodes and at most two mismatches to primers were retained. The screened high-quality reads were merged with FLASH (Magoč and Salzberg 2011) if overlaps were greater than 10 bp and had no more than 2 nucleotide mismatches. Operational taxonomic units (OTUs) were clustered using UPARSE (Edgar 2013) at a 97% identity level. Singleton and doubleton OTUs that may represent sequencing errors were removed. Taxonomy of the representative sequence for each OTU was assigned against the Silva database (Release 138, http://www.arb-silva.de) implementing the Ribosomal Database Project (RDP) Classifier (Wang et al. 2007). To equalize sequencing depth, each sample was rarefied to 46,577 reads (the lowest sequence number across all samples). Alpha and beta diversity indices were calculated with the ‘vegan’ package in R. Non-metric multidimensional scaling analysis and analysis of similarities were performed based on the Bray–Curtis dissimilarities.

### Metagenomic sequencing, assembly and functional characterization

Metagenomic DNA was extracted from 10 to 12 g of sediment samples for each experimental replicate as previously described (Cheng et al. 2023; Zhou et al. 1996). Briefly, biomass from 1 g samples was washed using 3.3 mL extraction buffer (100 mmol/L Tris–HCl [pH 8.0], 100 mmol/L sodium EDTA [pH 8.0], 100 mmol/L sodium phosphate [pH 8.0], 1.5 mol/L NaCl, 1% CTAB) and then centrifuged at 6000×g for 20 min at room temperature. The concentrated biomass was ground in liquid nitrogen. Proteinase K and SDS were added in sequence and incubated at 37 °C and 65 °C, respectively, followed by phenol–chloroform extraction. DNA was precipitated with 0.6 volume of isopropanol, washed with 70% ethanol and air dried. The PowerSoil DNA isolation kit (MoBio Lab) was used for DNA purification. DNA libraries were prepared without any amplification step. Metagenomic shotgun sequencing was performed on the Illumina HiSeq X-Ten platform, with 2 × 150-bp paired-end reads.

Metagenomic assembly, mapping, and binning were performed according to Xue et al. (2020) with modifications. Briefly, raw sequence data with > 10% undefined bases, > 40% low-quality bases and > 15 bases matching the adapters were removed with the metaWRAP-Read_qc module (Cheng et al. 2023; Uritskiy et al. 2018). The clean reads ranging from 17.16 to 21.88 Gb per sample were then assembled with metaSPAdes version v3.15.2 (Nurk et al. 2017). Ribosomal RNA reads were extracted from the metagenomes using a kmer strategy in BBDuk from the BBMap tool suite (Bushnell 2014) and were compared against the SILVA database using Qiime 2 (Bolyen et al. 2019). The approximate taxonomy of the bins was assigned against the Genome Taxonomy Database (GTDB) release 202. Prokka v1.14 (Seemann 2014) was used to predict the gene coding sequence with default Settings. The relative abundance of specific pathways/genes (*amoABC*) in metagenomes was assessed using the DiTing software (https://github.com/xuechunxu/DiTing; Xue et al. 2021). Functional gene relative abundance was calculated by read mapping and was indicated by copies per million mapped reads.

## Results

### Isotopic and chemical characterization

**Fig. 2:**
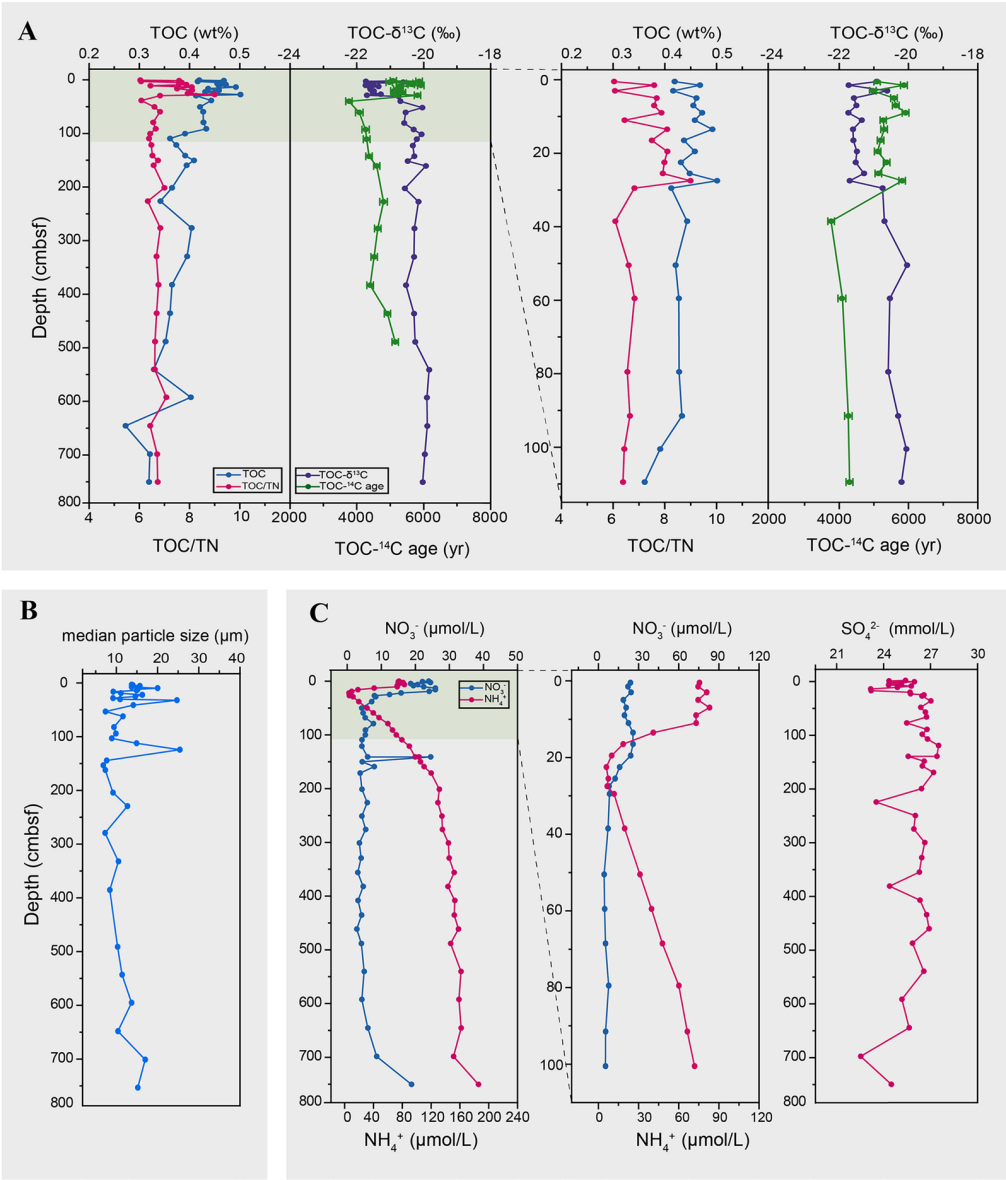
Vertical profiles of environmental and geochemical variables in the Mariana Trench sediment core. **A** Sedimentary TOC, TOC/TN, TOC-δ^13^C and TOC-^14^C, with the enlarged profiles for the top 111 cmbsf showing on the right. **B** Median particle size. **C** Profiles of porewater nitrate, ammonium and sulfate, with the enlarged profiles for nitrate and ammonium for the top 102 cmbsf showing on the right

Geochemical (δ^13^C and ^14^C ages of bulk OC and porewater nutrients), cell counting and microbial diversity analyses were performed on a 752 cm long sediment core collected from 10,816 mbsl from the Challenger Deep of the Mariana Trench (Fig. 1, Supplementary Fig. S1 and Table S2). The age of the core sediments was determined by measuring TOC-^14^C from depths between 0 and 490 cmbsf (Fig. 2A). The TOC-^14^C age of the upper 28 cmbsf ranged from 4970 to 5915 years, significantly older than the 37–40 cmbsf sample (3765 years). Below this, TOC-^14^C age increased with depth at 225–228 cmbsf (4800 years), declining at 381–384 cmbsf (4400 years), and then rising again at 487–490 cmbsf (5150 years). The TOC-δ^13^C values ranged between – 21.7‰ to – 19.8‰, increasing significantly from the upper 28 cmbsf to deeper layers (Wilcoxon Rank Sum test,* p* < 0.01; Fig. 2A). Thus, sedimentological and isotopic data (both δ^13^C and ^14^C ages) showed a clear shift in sedimentation patterns in the upper 28 cmbsf compared to the sediment below.

Total OC was on average higher in the upper 28 cmbsf (0.46 ± 0.02% wt%) and gradually decreased below this point (Fig. 2A), although there were episodic increases at discrete depths (e.g., 591–594 cmbsf). The TOC/TN ratios ranged from 6.0 to 9.0 also showing fluctuations in the top 28 cmbsf. The sediment median particle size ranged between 6.8 and 25.4 μm (Fig. 2B), indicative that silt (grain size of 4–63 μm) was the major component of the hadal subsurface sediments. Episodic increases in particle size were observed at 108–111 (14.8 μm) and 120–123 (25.4 μm) cmbsf. Furthermore, porewater analysis showed that NH_4_^+^ and NO_3_^–^ were relatively high at 15 and 21 cmbsf, respectively, before rapidly dropping at 28 cmbsf. Below this, NO_3_^–^ remained low, except for an abrupt increase at 140–143 cmbsf and > 700 cmbsf, while NH_4_^+^ gradually increased with depth (Fig. 2C). The concentration of SO_4_^2−^ was relatively constant throughout the core (Fig. 2C).

### Depth profile of microbial abundance

**Fig. 3:**
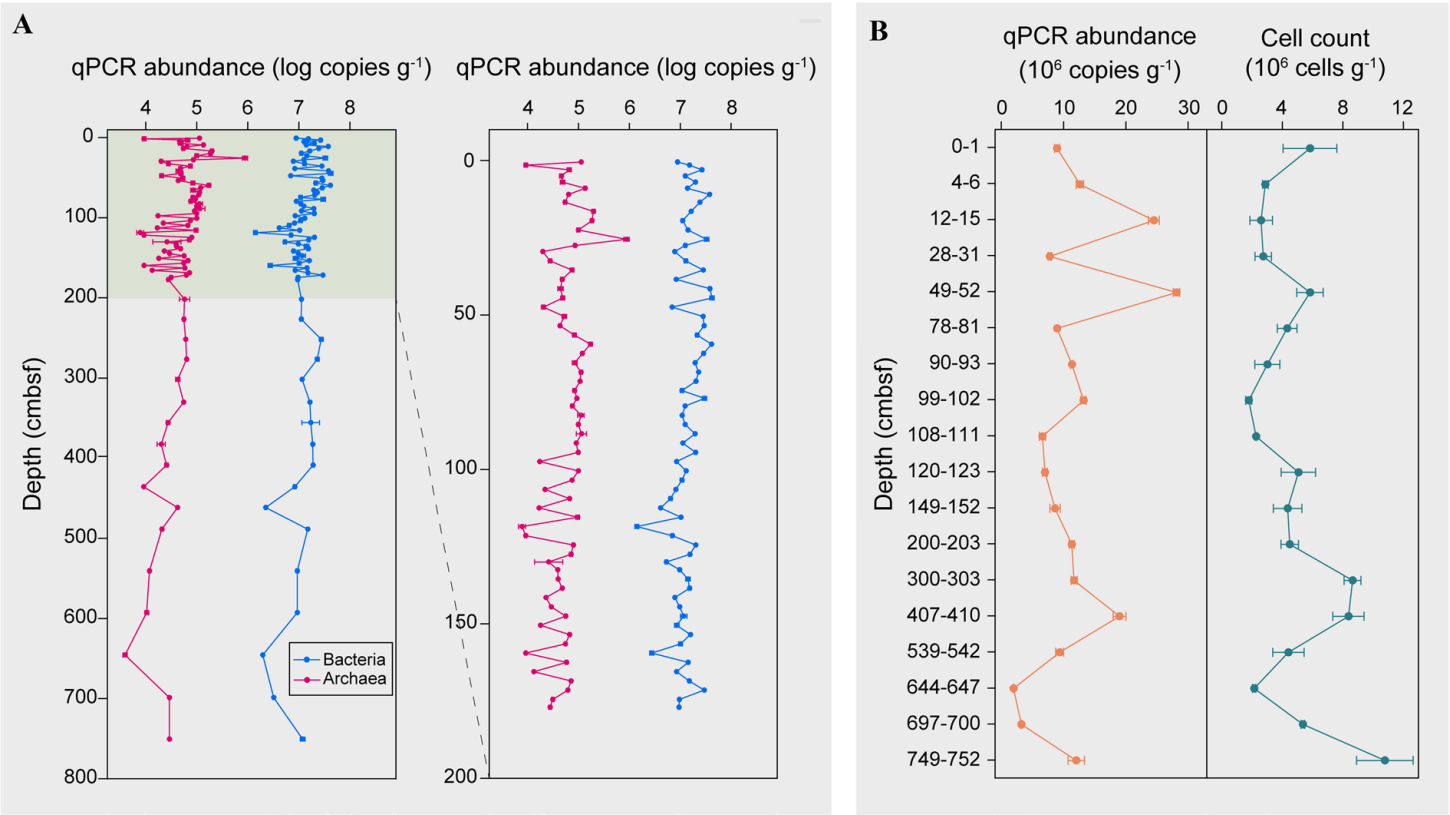
Prokaryotic abundance along the sediment column. **A** Log transformed bacterial and archaeal 16S rRNA gene copy numbers derived from qPCR, with the enlarged profiles for the top 200 cmbsf showing on the right. **B** Prokaryote cell counts measured at 18 sediment depths via flow cytometry and a parallel comparison with prokaryote 16S rRNA gene copy numbers derived from qPCR

To explore the effect of sedimentation and geochemical zonation on microbial communities, the abundance of prokaryotes was measured along a depth profile using qPCR and direct cell counting. The qPCR-estimated bacterial abundance (1.4 × 10^6^–4.2 × 10^7^ copies g^−1^ wet sediment) was approximately two to three orders of magnitude greater than archaea (3.9 × 10^3^–8.8 × 10^5^ copies g^−1^ wet sediment; Fig. 3A). However, the abundance of both bacteria and archaea was relatively consistent throughout the core. Direct counting showed a lower number of cells compared to gene quantification (Fig. 3B), similar to a previous study (Hiraoka et al. 2020). This may be attributed to biases caused by the different methods including efficiency of cell extraction, cell lysis and staining (Morono et al. 2009), and multiple 16S rRNA gene copy numbers per genome (Sun et al. 2013). Despite this, the results of both methods revealed similar depth trends, with some abundance fluctuation across the whole core, such as increased bacterial abundance at 49–52 cmbsf, 303–410 cmbsf and the bottom layer. The abundance of bacteria was positively related to the content of TOC, whilst that of archaea negatively correlated to NH_4_^+^ and SiO_3_^2−^ (Supplementary Fig. S2).

### Operational taxonomic unit occurrence and diversity pattern

**Fig. 4:**
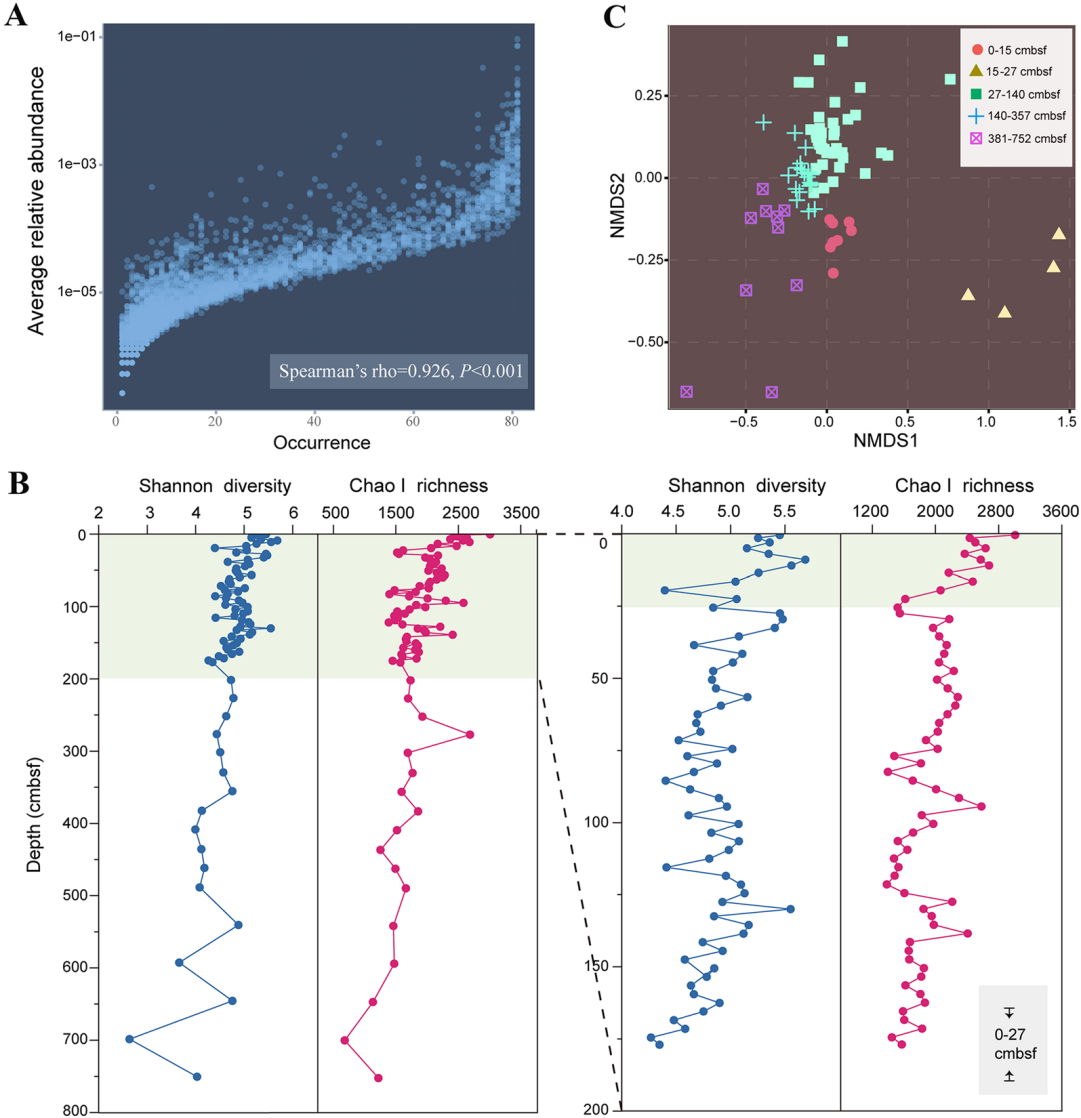
Abundance-occupancy and diversity pattern of OTUs. **A** Abundance-occupancy relationship based on all OTUs. Significant correlations were observed between relative abundance and number of occurring sites. **B** Alpha diversity along the sediment column shown as Shannon and Chao I indices, with the enlarged profiles for the top 200 cmbsf shown on the right. **C** Ordination of community using the non-metric multidimensional scaling based on Bray–Curtis dissimilarities

Amplicon sequencing of the 16S rRNA gene generated a total of 46,577 reads that were clustered into 8487 operational taxonomic units (OTUs) at an identity level of 97%. Seventy-five OTUs accounted for 54.0% of the total sequences whilst only 0.88% of the total OTU numbers occurred at all sediment layers. These included 72 bacterial OTUs and three archaeal OTUs that represented 51.7% and 2.3% of the total population, respectively. OTUs with higher relative abundance tended to be present at more depths (Fig. 4A). The alpha diversity represented by the Shannon index decreased from 0 to 27 cmbsf, before a steep rise at 28 cmbsf (Fig. 4B). The Chao I index decreased until 28 cmbsf, followed by a steep rise, likely reflecting discontinuous sedimentation between the top 27–28 cmbsf and deeper layers. Similar patterns were also observed in the Shannon evenness and phylogenetic diversity (Supplementary Fig. S3). Non-metric multidimensional scaling analysis showed an overall change in community composition with sediment depth (*P* < 0.05; Fig. 4C).

### Depth related community composition change

**Fig. 5:**
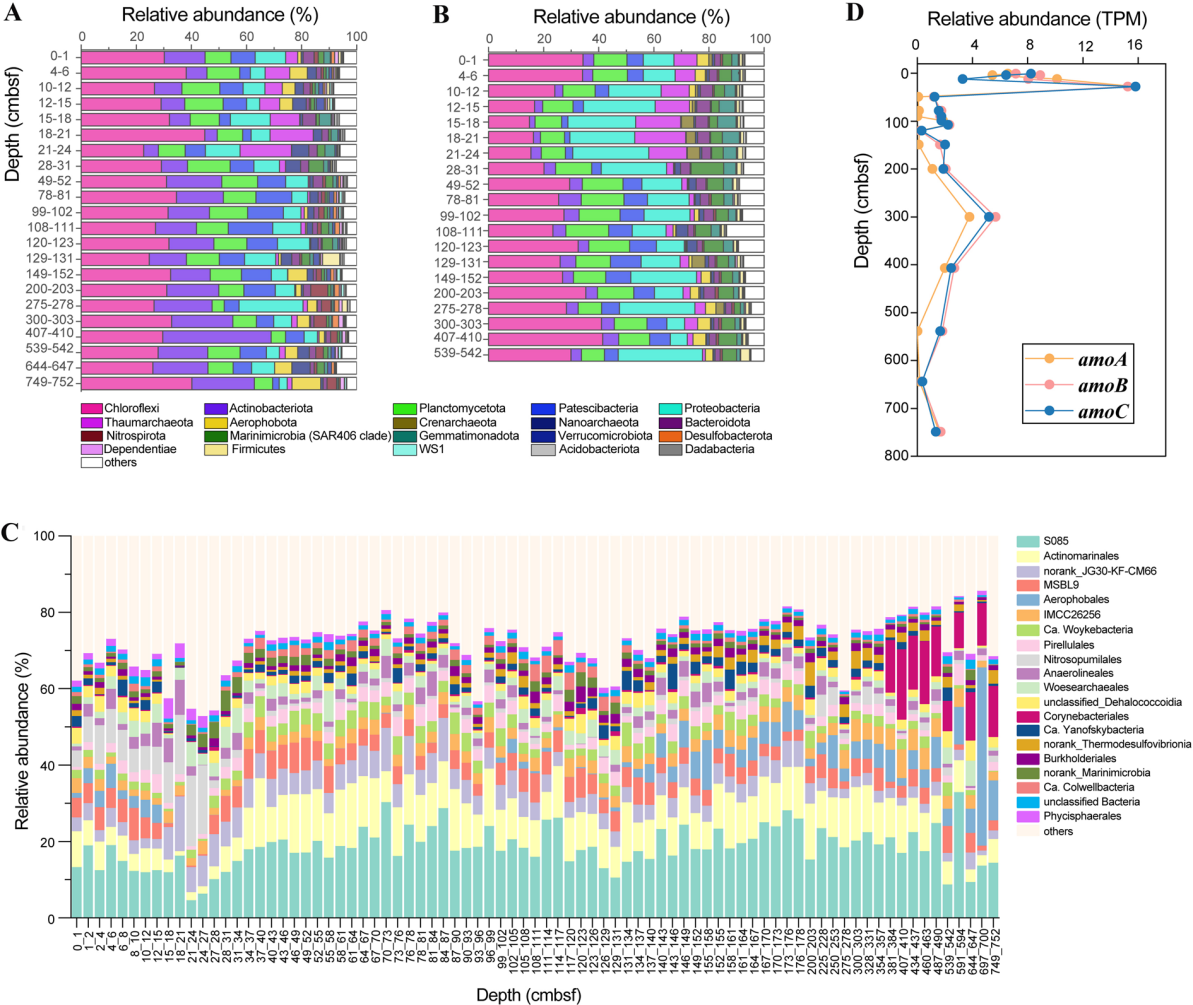
Microbial community composition along the sediment column. Depth profile of the top 20 most detected phyla as shown by 16S rRNA gene sequencing (**A**) and metagenomic sequencing (**B**), the top abundant 20 orders from amplicon sequencing (**C**) and the functional genes encoding ammonia monooxygenase (**D**)

Taxonomical assignment of 16S amplicons revealed the presence of 67 phyla across all sediment samples. *Chloroflexi* was the most abundant bacterial phylum accounting for 32.8% of all sequences (Fig. 5A), and 78.3% of the population represented by 25 OTUs across the whole core. *Actinobacteriota* (16.4%), *Planctomycetota* (10.8%), *Patescibacteria* (9.5%) and *Proteobacteria* (7.5%) were the next most abundant bacterial phyla (Fig. 5A). The dominant archaeal phyla were *Crenarchaeota* (2.5%) and *Nanoarchaeota* (2.4%). The dominance of these microbial groups was confirmed from the metagenomics analysis, although their relative abundance differed based on methods used (Fig. 5B). Both methods revealed a sharp shift in the microbial communities between the top 27 cmbsf and those in the sediments directly below (Fig. 5), most notably a higher population of *Nitrosopumilales* from the phylum *Thaumarchaeota* in the top ~ 27 cmbsf (Fig. 5C). *Nitrosopumilales* are aerobic and chemoautotrophic ammonia oxidizing archaea (Könneke et al. 2005). Correspondingly, genes encoding the ammonia monooxygenase (*amoABC*) showed high abundance in these sediment layers in the metagenomics analysis (Fig. 5D).

Dominant groups in the top sediment layers also included S085 of *Chloroflexi* and *Actinomarinales* of *Actinobacteria*, taxa that are preferentially distributed in oxic or relatively oxidized marine environments (Ghai et al. 2013; Vuillemin et al. 2020a). Both of them are unclassified at the genera level (Supplementary Fig. S4). However, unlike *Nitrosopumilales*, they tended to be more abundant in the deeper sediment layers (37–490 cmbsf), suggesting versatile respiration strategies. These two groups were the most abundant orders in the microbial community, jointly contributing 28.6% of the total community. OTUs from these two orders were present at all sediment depths, constituting 28.3% of the total community and 52.4% of the 75 OTUs. Below 27 cmbsf, community composition was relatively consistent except at 87–90 and 129–131 cmbsf, where there was a notable increase in *Firmicutes*; and from 99 to 117 and below 134 cmbsf, in which populations of *Aerophobota* also increased (Fig. 5A, B). Notably, *Aerophobota* also had a relatively high abundance in the top 15 cmbsf and was particularly abundant at 697–700 cmbsf.

### Extracellular enzyme activity

**Fig. 6:**
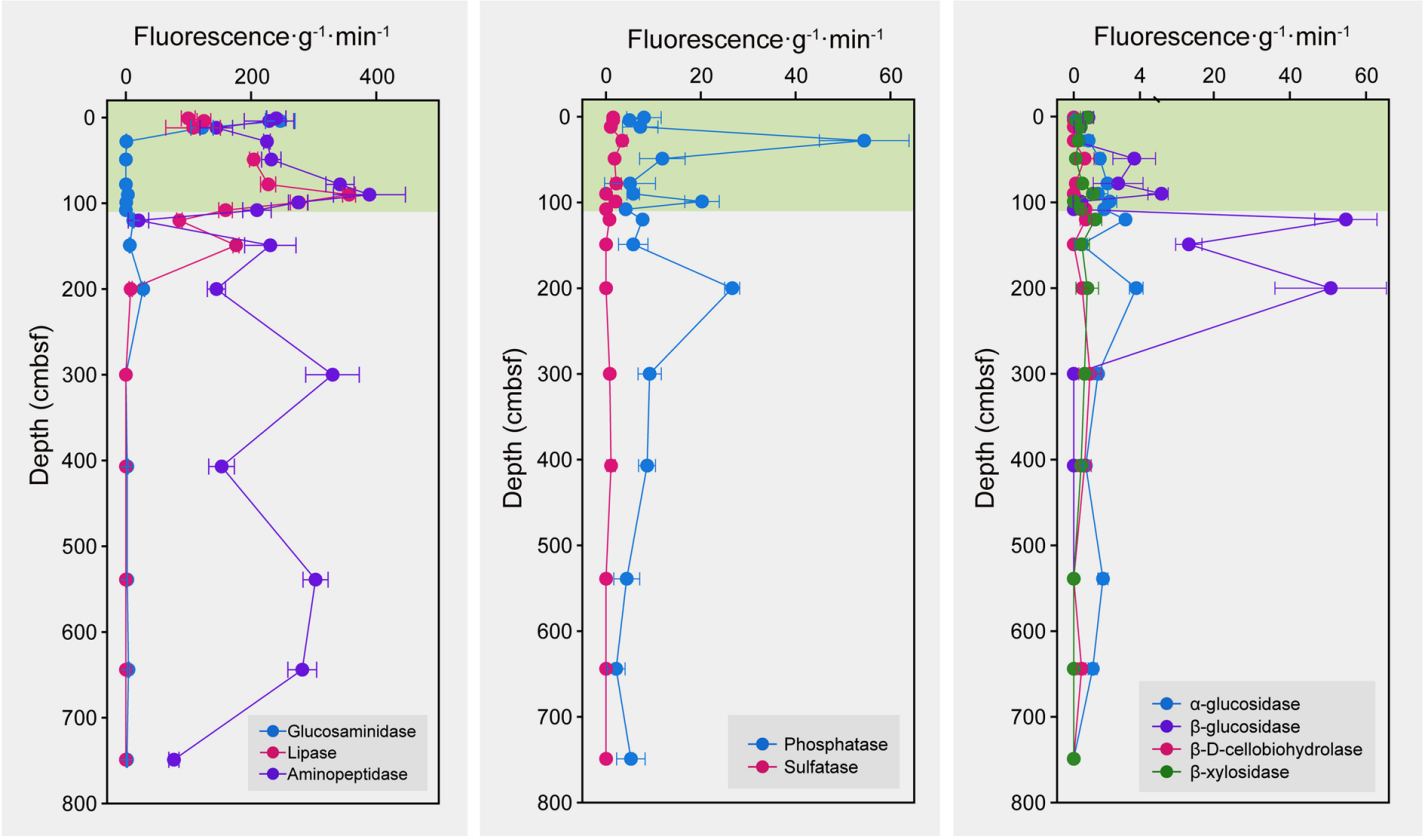
Extracellular enzyme activities in the sediment core. Activity was measured for nine different extracellular enzymes across 17 layers (*n* = 3). Extracellular enzyme activity was expressed as the fluorescence released from nonfluorescent substrates per gram of wet sediment per minute

To determine whether OC degradation capability changed throughout the core, the activities of a variety of extracellular enzymes targeting common macromolecules were measured, such as carbohydrates, proteins and lipids (Fig. 6). While activity of certain enzymes (sulfatases, *α*-glucosidases,* β*-D-cellobiohydrolase, *β*-xylosidase) was low or zero throughout the core, others showed large variations. Glucosaminidase activity was confined to the upper 15 cmbsf. Activity of lipases, aminopeptidases, phosphatases and* β*-glucosidases varied throughout the core, which suggests variations in the microbial population or differences in the type of organic matter deposited.

Spearman’s correlations between taxon abundance and extracellular enzyme activity showed that *β*-D-cellobiohydrolase, *α*-glucosidase and lipase activities were likely attributed to more diverse taxa than other enzymes (Supplementary Fig. S5). Although aminopeptidase had high activity, no significantly related taxa were observed. S085 and *Actinomarinales*, the most abundant taxa throughout the core, had positive correlations with *β*-xylosidase and lipase, and *β*-D-cellobiohydrolase, *α*-glucosidase and phosphatase, respectively. *Aerophobales,* with increased abundance in deeper sediment samples, showed a negative correlation with *α*-glucosidase.

## Discussion

### Rapid and discontinuous sedimentation in the Challenger Deep

Extensive analyses on subsurface microbial communities from the deepest oceanic site on Earth were performed on a ~ 750-cm long sediment core. The δ^13^C isotopic data and ^14^C ages showed clear shifts in sedimentation patterns with depth, especially in the upper 28 cmbsf. Older ^14^C ages and lower δ^13^C values in these top sediment layers may indicate sudden deposition of older material, likely from trench slopes. This is supported by the relatively younger ^14^C age of sinking particulate matter collected at 6000 m in the hadal zone of the southern Mariana Trench (Shan et al. 2020), indicating translocation of pre-aged OC to the Challenger Deep seabed. Below this depth, continuous deposition likely occurred between 37 and 228, and 381 and 490 cmbsf, but the younger material deposited between 275 and 381 cmbsf could potentially be due to another sudden sedimentation event. Previous geochemical studies also revealed OC-^14^C age offsets in other hadal zones (Schwestermann et al. 2021; Xiao et al. 2020; Xu et al. 2021), suggesting that noncontinuous sedimentation may be a common phenomenon in global hadal trenches. Frequently occurring earthquakes may act as an important driving force for mass-transport deposition events (Bao et al. 2018; Zhu et al. 2019).

The δ^13^C data fell within the range (– 19‰ to – 22‰) typical for marine origin of OC, indicating dominance of a marine-derived carbon source throughout the core, in line with a previous study of trench sediments (Luo et al. 2017), although the decreased δ^13^C values in the upper 28 cmbsf suggest a slight increase of terrestrial OC contribution. This differs from a sediment core collected from the western equatorial Atlantic which shows an alternating pattern of dominance of marine- and terrestrial-derived carbon, determined by paleoceanographic conditions (Freitas et al. 2020). The TOC content showed a decreasing trend with depth, in line with previous observations (Deng et al. 2020). However, this pattern is unexpected here, since the older material deposited in the top sediment layers may be devoid of carbon due to long-term degradation. It is speculated that these upper sediment samples (0–28 cmbsf) may have been transported laterally from sediments with relatively high carbon content, but the exact source needs further examination. Porewater analysis revealed decreased NO_3_^−^ in the top 20 cmbsf, indicating utilization of NO_3_^−^ as an electronic acceptor.

### Alteration of microbial abundance by sudden sedimentation events

Estimation of microbial abundance by qPCR and cell counting demonstrated an irregular depth profile, consistent with a recent report on prokaryotic abundance in the Kermadec and Atacama Trenches (0–30 cmbsf; Schauberger et al. 2021a). However, this is significantly different from the decreasing trend commonly seen in abyssal subsurface sediments, which is mainly caused by a decline in energy sources (Kallmeyer et al. 2012). In the Challenger Deep sediment core studied here, the TOC content decreased only slightly with depth, with any fluctuations likely due to episodic deposition events. Thus, rapid discontinuous sedimentation may introduce bioavailable marine-origin OC that can be buried in the hadal subsurface and fuel the growth of prokaryotic life therein. This is in line with the observed positive correlation between bacterial abundance and TOC concentration and supported by the detectable enzymatic activity of leucyl aminopeptidase, capable of degrading amino acids and amino sugar that have high bioavailability (Davis and Benner 2005). Different enzymes differed in their vertical distribution pattern, indicating that the microbial abundance may also be controlled by the composition of organic carbon, which warrants further investigation. In situ growth of bacterial groups in the deep subseafloor has been previously attributed to specific metabolic capacity (Vuillemin et al. 2020b) and redox zonation (Zhao et al. 2020). By establishing a link between geological processes and energy sources, this study provides new insights into the mechanisms sustaining growth of prokaryotes likely to occur in the deepest parts of the ocean beneath the seafloor.

### Compositional change reflecting redox conditions and sedimentation events

Despite the likelihood of mass wasting deposition, microbial community composition has recently been reported to be largely similar across trenches, caused by the availability of electron acceptors (Schauberger et al. 2021b). *Nitrosopumilales* are aerobic and chemoautotrophic ammonia-oxidizing archaea predominately distributed in global marine surface sediments (Könneke et al. 2005; Martens-Habbena et al. 2009; Qin et al. 2020). This population, as well as the genes encoding ammonia monooxygenase, had high abundance in the top 27 cmbsf, especially 15–27 cmbsf, which might account for the rapid drop of ammonium. Thus, the high abundance of *Nitrosopumilales* in these top sediment layers was likely due to the availability of oxygen, which penetrated into the shallow sediment layers (∼20 cm; Glud et al. 2013), rather than allochthonous cells transported by sudden sediment deposition. In deeper anoxic sediments, the presence and activity of *Nitrosopumilales* may be supported by the oxygen produced by itself (Kraft et al. 2022).

However, evidence for introduction of allochthonous cells was found by the relatively higher abundance of *Aerophobota* in the top 15 cmbsf than in the sediments immediately below. *Aerophobota* had low abundance between ~ 15 and ~ 130 cm and then increased at deeper depths. Being abundant in surface shallow sediments is unusual for *Aerophobota* because members of this phylum have been found ubiquitously distributed in anoxic subseafloor sediments (D’Hondt et al. 2019; Orsi 2018; Zhang et al. 2021). It is hypothesized that *Aerophobota* in the top 15 cmbsf resulted from the episodic deposition of older material from the slope. Comparison of *Aerophobota* sequences between the surface and deep sediment samples showed they belonged to the same OTU, which supports this hypothesis. These data suggest that caution must be applied when analyzing sediments at shallow depths in trenches, as these populations may be present due to sudden rather than gradual sediment deposition and this may not accurately reflect the resident microbial assemblages.

### Predominance of specific bacterial groups throughout the sediment core

In addition to the above noted community changes, many abundant OTUs and some bacterial clades were found, represented by S085 of *Chloroflexi* and *Actinomarinales of Actinobacteriota*, at all sediment depths. Previous studies have reported the dominance of *Chloroflexi* in hadal trench surface sediments with S085 being one of the most abundant orders (Liu et al. 2022; Wu et al. 2023). Results presented here expand these results by showing that S085 was also abundant in the hadal subseafloor. Although the genetic potential of S085 is poorly understood, the preliminary analysis of the genome sequence of RBG-16-64-32 in the GTDB database (the genome has a short ribosomal rRNA sequence annotated as S085 in the Silva database) revealed the presence of a gene encoding nitrate oxidoreductase. This together with the detectable nitrate concentration throughout the core suggests that this group may respire nitrate under anoxic conditions. *Actinomarinales* is mainly found at the ocean surface and has a notably small genome and cell size (Ghai et al. 2013; López-Pérez et al. 2020). Sequence representatives of the most abundant *Actinomarinales* OTUs were close to sequences retrieved from deep-sea sediments rather than sequences from pelagic environments, suggesting niche partitioning in this lineage (Supplementary Fig. S6). These bacterial groups might gain bioavailable carbon source via input from the episodic sedimentation events. This warrants further verification by genomic and isotope probing analyses.

It was assumed that the dominance of specific OTUs at all sediment depths may have decreased the depth-related community heterogeneity. To confirm this, a comparison was made of the cross-depth community dissimilarities in this study with those derived from four sediment cores in the Baltic Sea-North Sea transition (Deng et al. 2020) and ten from the South China Sea (Zhang et al. 2021) with similar sampling depths (5–8 mbsf). The results showed substantially lower depth-related heterogeneity in the Mariana Trench (Supplementary Fig. S7) compared with these other sites. Thus, changes in redox conditions and organic matter availability significantly affect the subseafloor microbiosphere in the Mariana Trench, likely acting as a constraint driving converged community compositions at different depths.

## Conclusion

Our results suggest that episodic downslope mass-transport events disrupted continuous sediment deposition at the deepest section of the Mariana Trench. These processes introduced non-indigenous microbes and resulted in accumulation of OC and alteration of redox conditions, which play a significant role in shaping the subseafloor community composition and the erratic abundance and activity profiles with depth. The findings suggest that hotspots of carbon turnover may not be constrained to the hadal surface sediments, emphasizing the role of hadal trenches in the global carbon cycle. Our results expand current knowledge on the hadal benthic biosphere by showing a significant impact of sudden sedimentation processes, providing novel insights into life in deep subsurface sediments.

### Supplementary Information

Below is the link to the electronic supplementary material.

Supplementary file1 (DOCX 1859 KB)

## Data Availability

The 16S amplicon datasets generated during the current study are available in the National Omics Data Encyclopedia under the accession number PRJCA008837. The metagenome data are from our recent work (Cheng et al. 2023) and deposited in the NCBI database under the accession number PRJNA957232.
