# Supplementary material for: A unique subseafloor microbiosphere in the Mariana Trench driven by episodic sedimentation
Source: Mar Life Sci Technol. 2024 Jan 23;6(1):168–81. doi: 10.1007/s42995-023-00212-y (PMC10902237; doi:10.1007/s42995-023-00212-y)
Supplement: Supplementary file 1 — Supplementary file1 (DOCX 1859 KB) [file 42995_2023_212_MOESM1_ESM.docx]

**Supplemental Information for:**

**A unique subseafloor microbiosphere in the Mariana Trench driven by episodic sedimentation**

Jiwen Liu^1,2,3†^, Da-Wei Li^2,4^^†^, Xinxin He^1,3†^, Ronghua Liu^1,3^, Haojin Cheng^1,3^, Chenglong Su^4^, Mengna Chen^4^, Yonghong Wang^5^, Zhongsheng Zhao^6^, Hanyue Xu^4^, Zhangyu Cheng^4^, Zicheng Wang^4^, Nikolai Pedentchouk^7^, David J. Lea-Smith^8^, Jonathan D. Todd^8^, Xiaoshou Liu^1,3^*, Meixun Zhao^2,4^*, Xiao-Hua Zhang^1,2,3^*


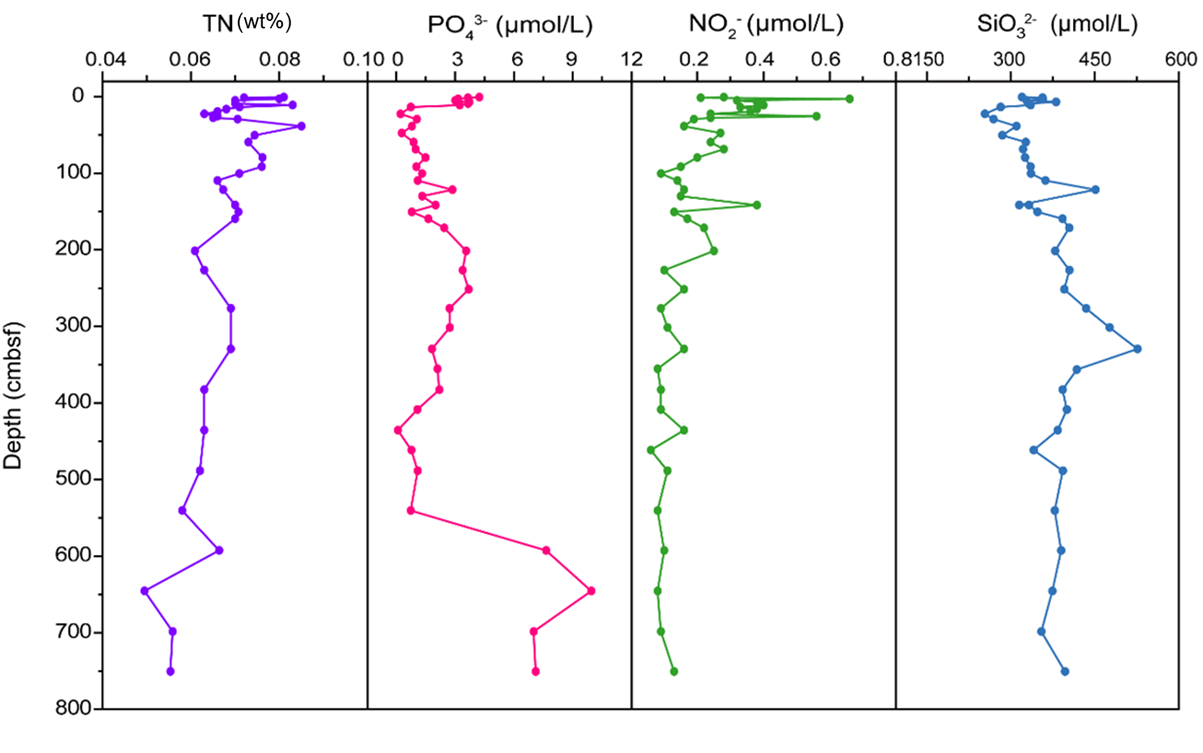


Fig. S1. Vertical profiles of sedimentary total nitrogen (TN) content and porewater nutrient concentrations (PO_4_^3-^, NO_2_^-^ and SiO_3_^2-^) throughout the core.

**
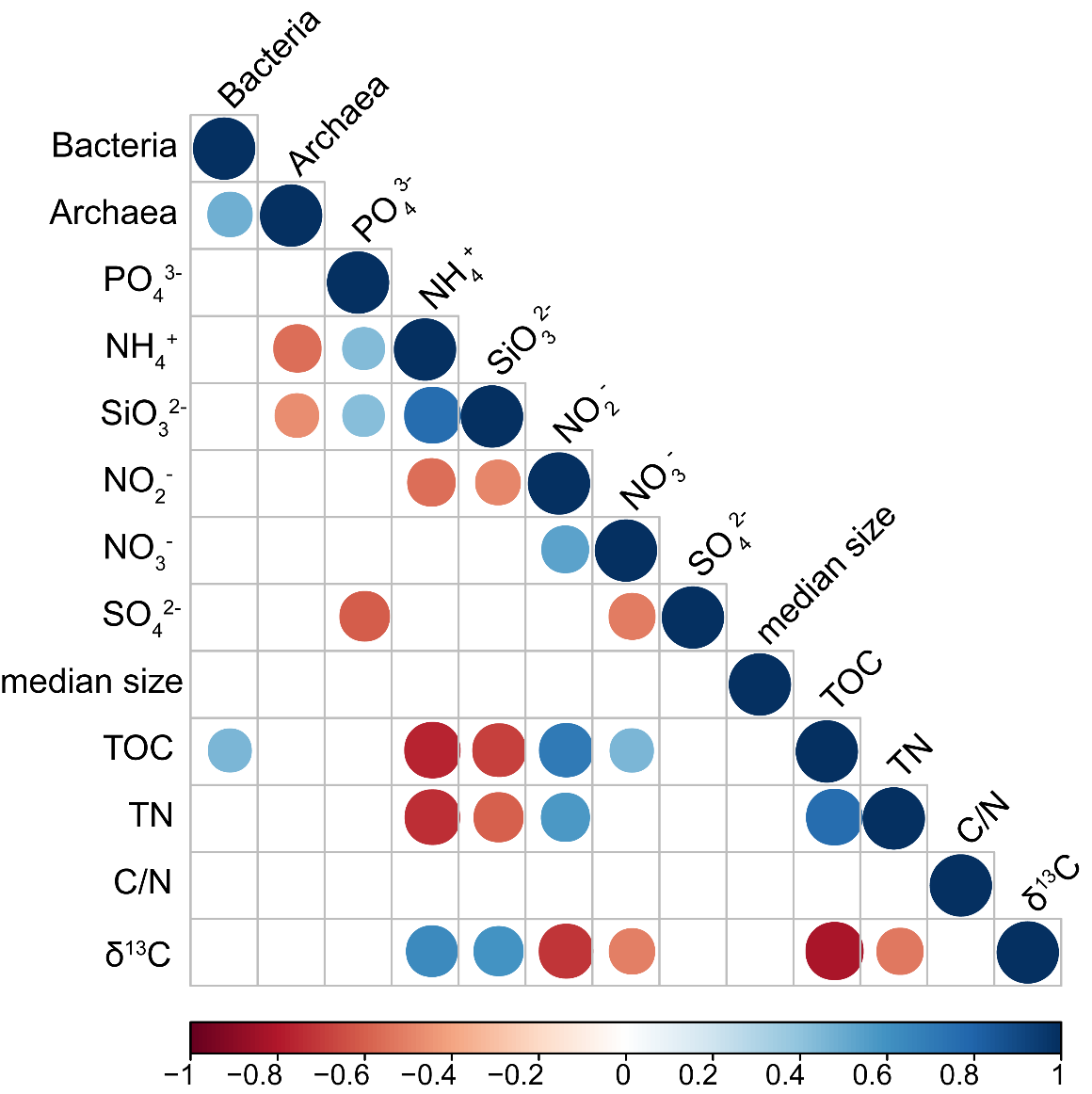
**

Fig. S2. Correlation matrix between microbial abundance and environmental factors. Insignificant values (p<0.01) were shown in blank.

**
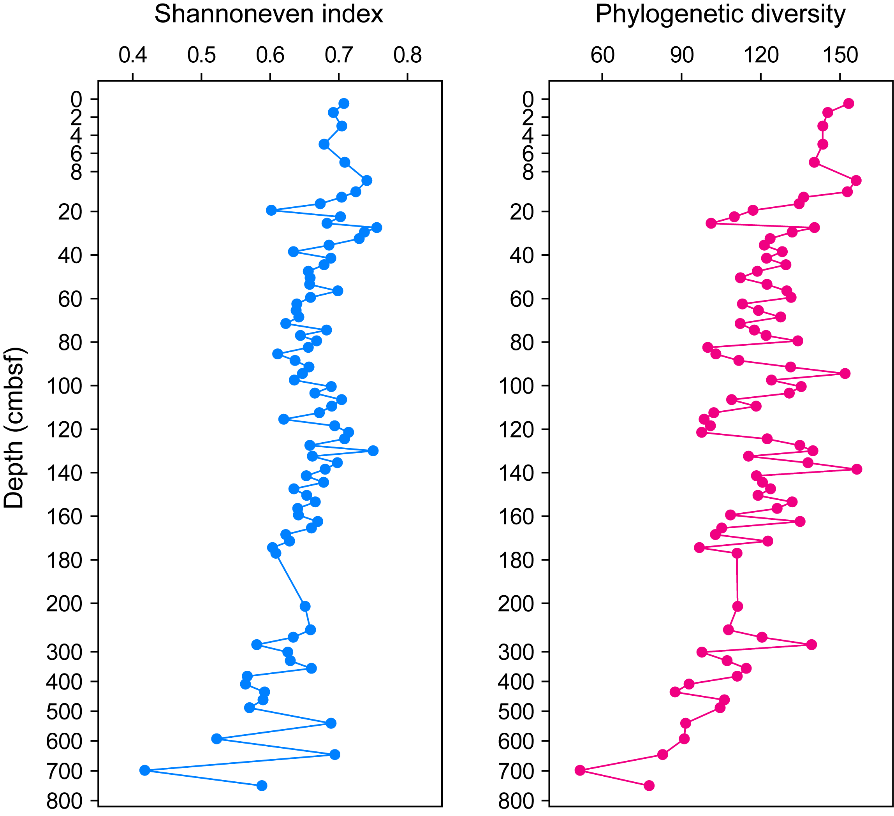
**

Fig. S3. Vertical profiles of Shannon evenness and phylogenetic diversity along the sediment column.


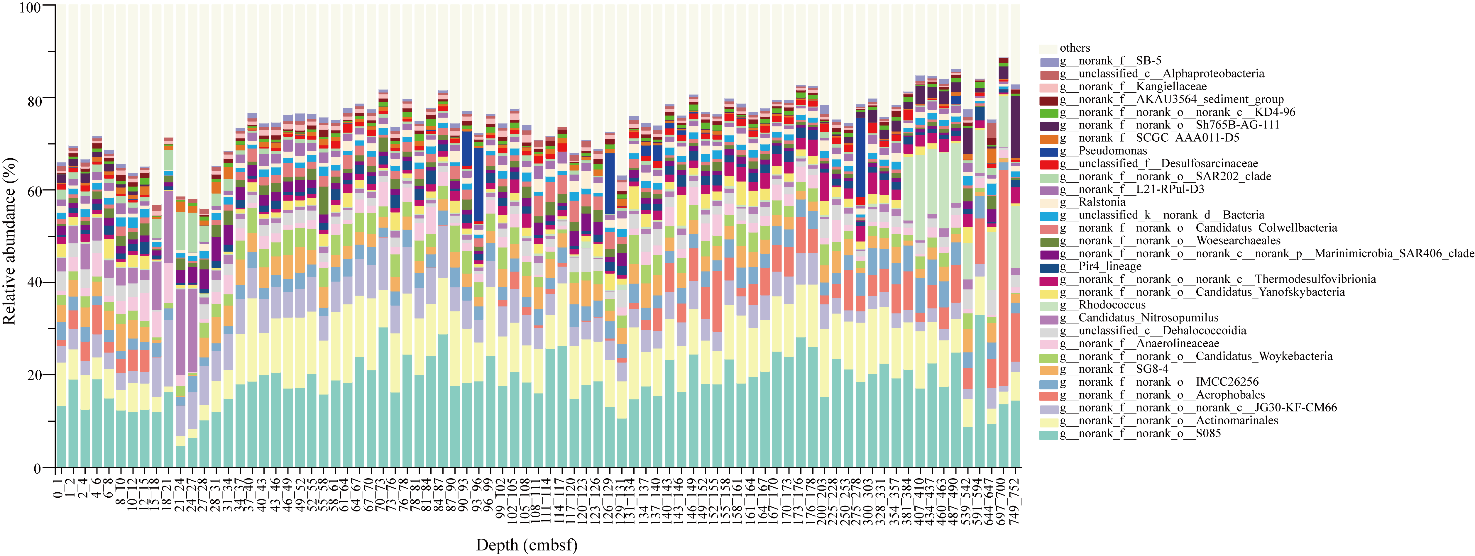


Fig. S4. Relative abundance of the top abundant genera along the sediment column


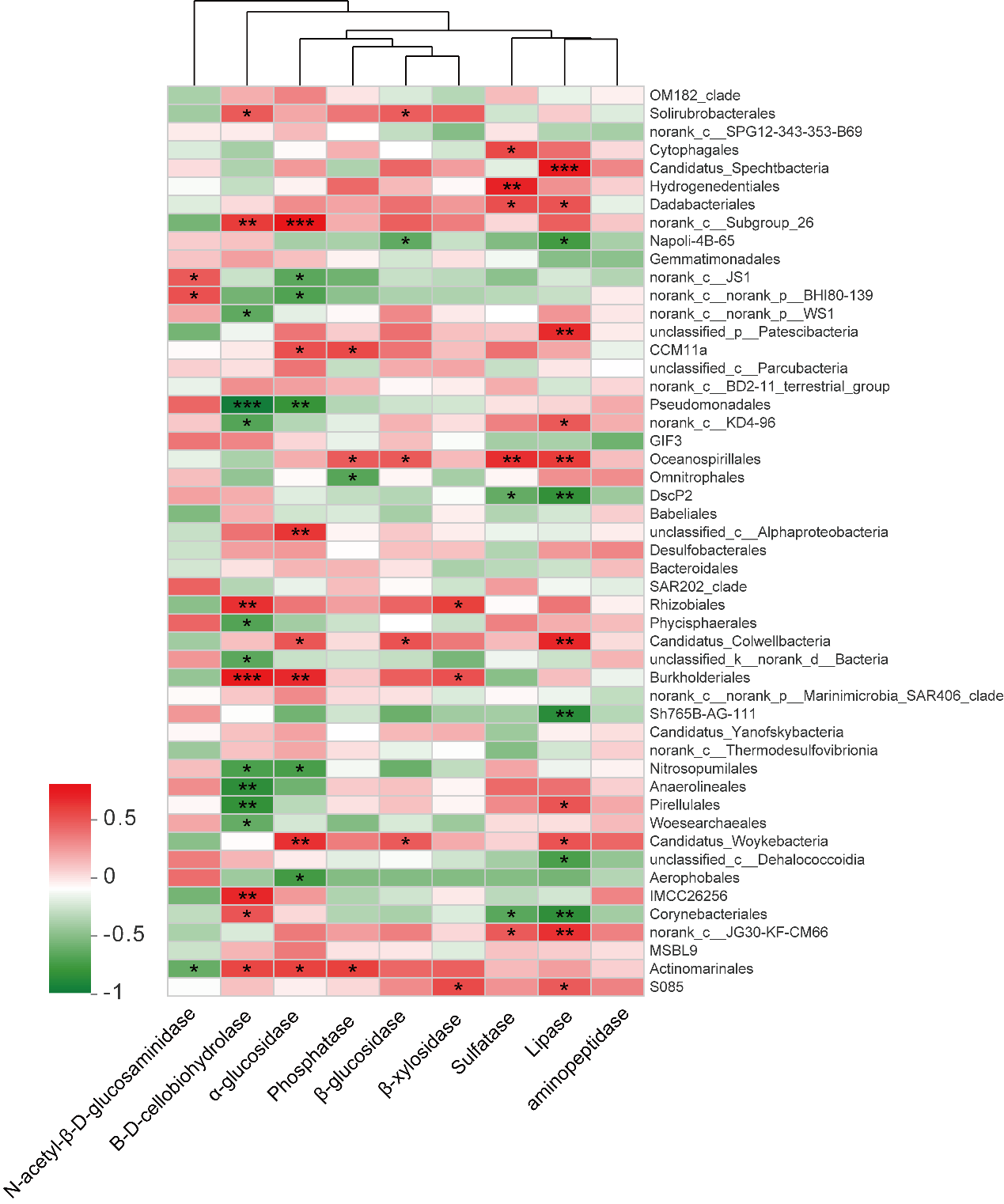


Fig. S5. Heat map showing the correlation between dominant orders and extracellular enzymatic activity. ** indicates a *p* value of <0.01 and *** indicates a *p* value of <0.001.


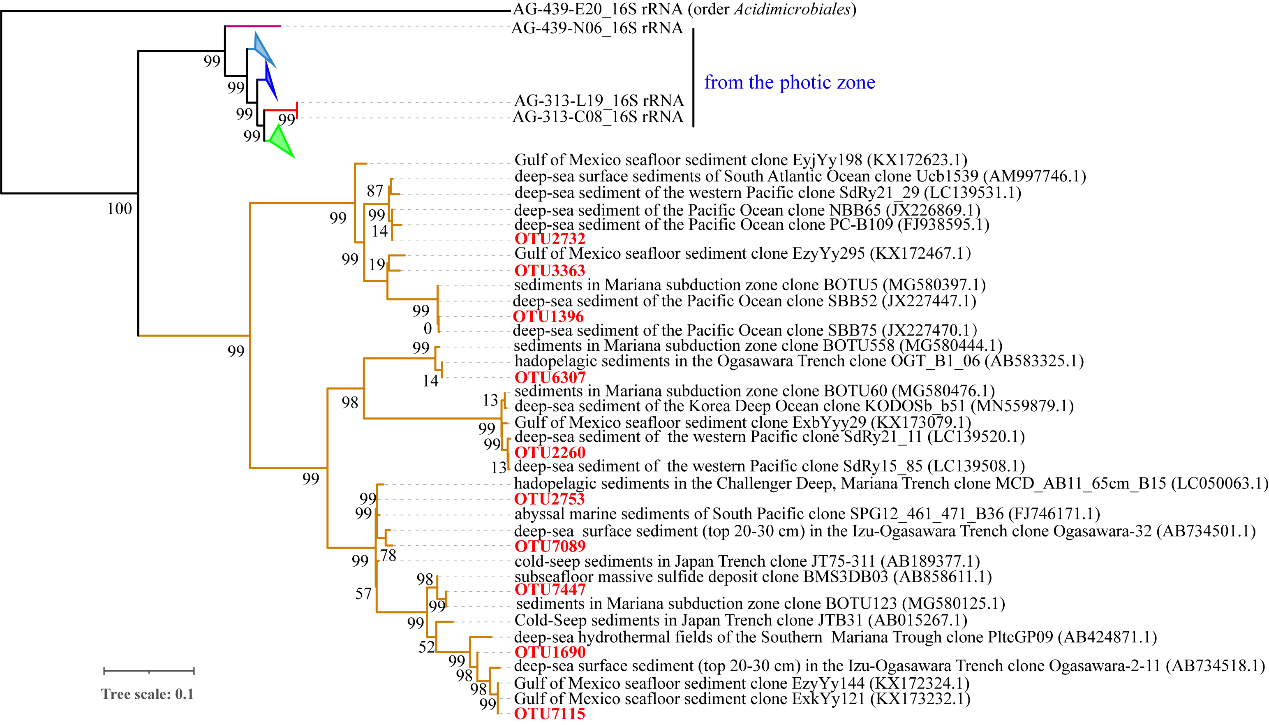


Fig. S6. Maximum likelihood phylogenetic tree based on the most abundant *Actinomarinales* OTUs in the deep-sea sediments (marked in red font) and closed related 16S rRNA gene sequences retrieved from the NCBI. The results showed that the *Actinomarinales* OTUs in the deep-sea sediments formed a distinct cluster with the members in the photic zone. The 16S rRNA gene annotated in genome AG-439-E20 from order *Acidimicrobiales* was used as an outgroup. Bar, 0.1 substitutions per nucleotide position.

**
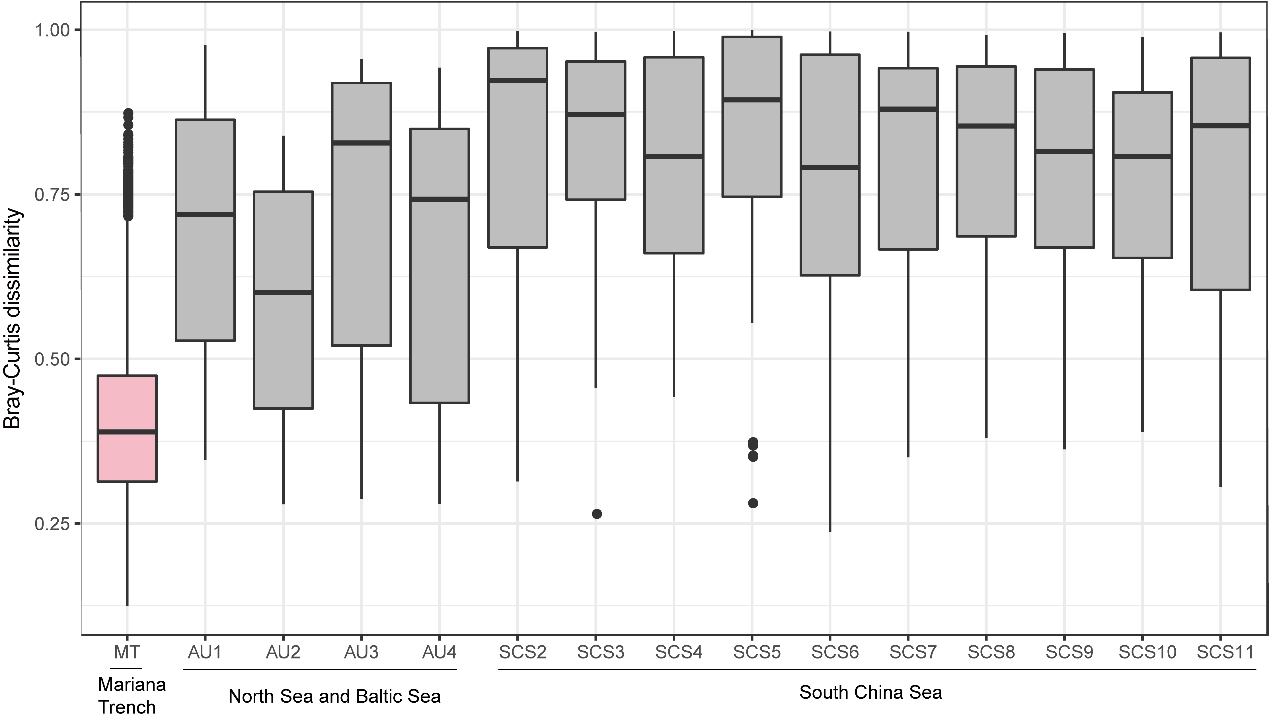
**

Fig. S7. Comparison of cross-depth Bray-Curtis dissimilarities between sediment core in the Mariana Trench of this study and those from the Baltic Sea-North Sea transition and the South China Sea with similar core lengths. Prokaryotic communities were used for the calculation in the Mariana Trench and the South China Sea, while only bacterial communities were used in the Baltic Sea-North Sea transition.

**Supplementary Tables**

Table S1. Summary of studies on microbial populations in hadal trench sediments.

|  | Location: | Water depth (m) of sample collection | Length of core (cmbsf) | Sample ID and discrete depth analysed (cmbsf) | Organic and nitrogen geochemistry | Porewater nutrients | Other factors | Sediment particle sizes | Cell counting | 16S rRNA gene (SSU rRNA genes) sequencing | Metagenome samples | Transcriptome samples |
| --- | --- | --- | --- | --- | --- | --- | --- | --- | --- | --- | --- | --- |
| This study | Challenger Deep | 10,816 | 752 | Total of 81 samples:  See Table S2 | TOC, TN, δ^13^C, ^14^C age | NO_3_^-^, NO_2_^-^, NH_4_^+^, PO_4_^3-^, SiO_4_^2-^, SO_4_^2-^ | extracellular enzyme activities | yes | yes | 81 | 22 | / |
| Cui et al., 2019^1^ | Challenger Deep | 5,481-10,911 | 15-66 | Total of 95 samples:  3-17 depths for one core | / | / | / | / | / | 95 | / | / |
| Chen et al., 2021^2^ | Challenger Deep | 10,840 | 21 | 0-5, 5-10,10-14 | TOC, TN, δ^13^C, δ^15^N, | NO_3_^-^, NO_2_^-^, NH_4_^+^, PO_4_^3-^, SO_4_^2-^, | / | / | / | / | 11 | / |
| Liu et al., 2020^3^ | Mariana (Challenger Deep) and Mussau Trenches | Mariana: 10,853  Mussau:  7,011 | 21 | Total of 18 samples, nine depths for one core:  0-2, 2-3, 3-4, 4-5, 5-6, 6-7, 7-8, 8-9, 9-10 | / | / | / | / | / | 18 | / | / |
| Liu et al., 2022^4^ | Challenger Deep | 10853 | 21 | 0-2, 2-3, 3-4, 4-5, 5-6, 6-7, 7-8, 8-9, 9-10 | / | / | / | / | / | 9 | 9 | / |
| Zhou et al., 2022^5^ | Challenger Deep | 5,400-10,911 | 3-66 | Total of 37 samples:  1-5 depths for one core | TOC, TN | NO_3_^-^, NO_2_^-^, NH_4_^+^ | Dissolved O_2_ | / | / | / | 37 | 3 |
| Nunoura et al., 2018^6^ | Challenger Deep | 10,257 | ~123 | 5, 12.5, 65, 123 | TOC, TN, ^13^C/^12^C, ^15^N/^14^N, and ^18^O/^16^O | NO_3_^-^, NO_2_^-^, NH_4_^+^, PO_4_^3-^, SO_4_^2-^, | / | yes | / | 4 | / | / |
| Li et al., 2018^7^ | Southern Mariana  Trench | 4,500 | 151 | 30, 94, 151 | TC, TOC, TIC | / | / | / | / | 3 | / | / |
| Hiraoka et al., 2020^8^ | Mariana, Izu-Ogasawara and Japan Trenches | Mariana: 4,700-10,902  Izu-Ogasawara:5,257-9,776  Japan: 7,963 | Mariana:20-44  Izu-Ogasawara:15-155  Japan: 44 | Total of 92 samples:  4-10 depths for one core | TOC, TN, ^13^C, ^15^N | NO_3_^-^, NO_2_^-^, NH_4_^+^, PO_4_^3-^ | Dissolved O2 | / | yes | 92 | / | / |
| Peoples et al., 2019^9^ | Mariana and Kermadec Trenches | Mariana: 6,844-7,942;  Kermadec: 6,011- 9,177 | Mariana: 10  Kermadec: 5-12 | Mariana: 10 depths (intervals=1) for one core;  Kermadec: 3-4 depths for one core | / | / | Isolation of bacteria | / | / | 72 | / | / |
| Rastelli et al., 2019^10^ | Izu-Bonin Trench | 9,776 | 155 | 0-1, 10-15, 15-20, 30-35, 45-55, 55-65, 95-105, 115-125, and 145-155 | TOC | NH_4_^+^, NO_3_^-^ | pH, Dissolved O_2_, extracellular enzyme activities | / | Yes | 9 | / | / |
| Schauberger et al., 2020^11,12^; Thamdrup et al., 2021^13^ | Kermadec and Atacama Trenches | Kermadec Trench: 6,080-10,010;  Atacama Trench: 2,560-7,770 | Kermadec Trench: 4-30;  Atacama Trench: 6-25 | <30 (not shown in detail) | TOC | NH_4_^+^, NO_2_^-^  Mn^2+^, Fe^2+^ | O_2_ | / | Yes | Atacama Trench: 395;  Kermadec Trench: 59 | / | / |
| Jing et al. 2022^14^ | Mariana Trench | abyssal-hadal transition zone: 5,455 (A); 5,481 (B); 5,482 (D114); 6,014， (D119); 6,333 (D144); 6,676 (D146); 6,693 (D147); 6,707 (D120) | A: 0-6; B: 0-18; D114: 0-12;  D119: 0-12;  D144: 0-18; D146:0-6;  D147:0-12;  D120:0-6 | sliced at 6 cm intervals, total 15 samples | TP, TN, TC  ^13^C, ^15^N | NO_3_^-^, NH_4_^+^ | Moisture content | / | / | / | 15 | / |
| Wang et al. 2022^15^ | Mariana and Mussau trenches | Mariana Trench: 10,853; Mussau Trench: 7,011 | 10 | 0-2 cm, 2-10 cm was sliced at 1cm intervals | / | / | / | / | / | Mariana Trench: 9; Mussau Trench: 9 | / | / |
| Fu et al. 2020^16^ | Southern Yap Trench | 6,501 | 21.5 | Total of 16 samples:  0.5,1.5, 2.5, 3.5, 4.5, 5.5, 6.5, 7.5, 8.5, 9.5, 11, 13, 15, 17, 19,  21.5 | TOC, TN, ^13^C, ^15^N | / | specific surface area | yes | / | 16 | / | / |
| He et al. 2022^17^ | Challenger Deep | 10,898 | 25 | not shown in detail | / | / | / | / | / | 1 | 1 | / |
| Wang et al 2022^18^ | Mariana Trench | 6,957–10,918 | surface sediment:0-4 | One sample per site, total 8 samples | TC, TN, TP | NH_4_^+^, NO_3_^-^ | / | / | / | 8 | / | / |
| Zhang et al 2022^19^ | Mariana Trench | 6,010 | not shown in detail | 3 subsamples, not shown in detail | / | / | / | / | / | 3 | 3 | / |
| Su et al. 2022^20^; Zhang et al. 2018^21^ | Yap Trench | Three surface sediment samples: 4,435; 4,568; 6,578 | 4,435: 0-2;  4,568: 0-2;  6,578: 0-5 | 4,435: 0-2;  4,568: 0-2;  6,578: 0-5 | / | NH_4_^+^, NO_2_^-^, PO_4_^3-^ | pH, Temperature | / | / | 3 | 3 | / |

Reference:

1. Cui G, Li J, Gao Z, Wang Y (2019) Spatial variations of microbial communities in abyssal and hadal sediments across the Challenger Deep. Peer J 7:e6961

2. Chen P, Zhou H, Huang Y, Xie Z, Zhang M, Wei Y, Li J, Ma Y, Luo M, Ding W (2021) Revealing the full biosphere structure and versatile metabolic functions in the deepest ocean sediment of the Challenger Deep. Genome Biol 22:207

3. Liu R, Wang Z, Wang L, Li Z, Fang J, Wei X, Wei W, Cao JW, Wei Y, Xie Z (2020) Bulk and active sediment prokaryotic communities in the Mariana and Mussau Trenches. Front Microbiol 11:1521

4. Liu R, Wei X, Song W, Wang L, Cao J, Wu J, Thomas T, Jin T, Wang Z, Wei W (2022) Novel Chloroflexi genomes from the deepest ocean reveal metabolic strategies for the adaptation to deep-sea habitats. Microbiome 10:75

5. Zhou Y-L, Mara P, Cui G-J, Edgcomb VP, Wang Y (2022) Microbiomes in the Challenger Deep slope and bottom-axis sediments. Nat Commun 13:1515

6. Nunoura T, Nishizawa M, Hirai M, Shimamura S, Harnvoravongchai P, Koide O, Morono Y, Fukui T, Inagaki F, Miyazaki J (2018) Microbial diversity in sediments from the bottom of the Challenger Deep, the Mariana Trench. Microbes Environ 33:186–194

7. Li Y, Cao W, Wang Y, Ma Q (2019) Microbial diversity in the sediments of the southern Mariana Trench. J Oceanol Limnol 37:1024–11029

8. Hiraoka S, Hirai M, Matsui Y, Makabe A, Minegishi H, Tsuda M, Rastelli E, Danovaro R, Corinaldesi C, Kitahashi T (2020) Microbial community and geochemical analyses of trans-trench sediments for understanding the roles of hadal environments. ISME J 14:740–1756

9. Peoples LM, Grammatopoulou E, Pombrol M, Xu X, Osuntokun O, Blanton J, Allen EE, Nunnally CC, Drazen JC, Mayor DJ (2019) Microbial community diversity within sediments from two geographically separated hadal trenches. Front Microbiol 10:347

10. Rastelli E, Corinaldesi C, Dell’Anno A, Tangherlini M, Lo Martire M, Nishizawa M, Nomaki H, Nunoura T, Danovaro R (2019) Drivers of bacterial α-and β-diversity patterns and functioning in subsurface hadal sediments. Front Microbiol 10:2609

11. Schauberger C, Glud RN, Hausmann B, Trouche B, Maignien L, Poulain J, Wincker P, Arnaud-Haond S, Wenzhöfer F, Thamdrup B (2021) Microbial community structure in hadal sediments: high similarity along trench axes and strong changes along redox gradients. ISME J 15:3455–13467

12. Schauberger C, Seki D, Cutts EM, Glud RN, Thamdrup B (2023) Uniform selective pressures within redox zones drive gradual changes in microbial community composition in hadal sediments. Environ Microbiol 25:1594-1604

13. Thamdrup B, Schauberger C, Larsen M, Trouche B, Maignien L, Arnaud-Haond S, Wenzhöfer F, Glud RN (2021) Anammox bacteria drive fixed nitrogen loss in hadal trench sediments. Proc Natl Acad Sci USA 118:e2104529118

14. Jing H, Xiao X, Zhang Y, Li Z, Jian H, Luo Y, Han Z (2022) Composition and ecological roles of the core microbiome along the Abyssal-Hadal transition zone sediments of the Mariana Trench. Microbiol Spectr 10:e01988-21

15. Wang Z, Wang L, Liu R, Li Z, Wu J, Wei X, Wei W, Fang J, Cao J, Wei Y, Xie Z (2022) Community structure and activity potentials of archaeal communities in hadal sediments of the Mariana and Mussau trenches. Mar Life Sci Technol 4:150-161

16. Fu L, Li D, Mi T, Zhao J, Liu C, Sun C, Zhen Y (2020) Characteristics of the archaeal and bacterial communities in core sediments from Southern Yap Trench via in situ sampling by the manned submersible Jiaolong. Sci Total Environ 703:134884

17. He L, Huang X, Zhang G, Yuan L, Shen E, Zhang L, Zhang X-H, Zhang T, Tao L, Ju F (2022) Distinctive signatures of pathogenic and antibiotic resistant potentials in the hadal microbiome. Environ Microbiome 17:19

18. Wang F, Zhang Y, Jing H, Liu H (2022) Spatial variation and metabolic diversity of microbial communities in the surface sediments of the Mariana Trench. Front Microbiol 13:1051999

19. Zhang X, Wu K, Han Z, Chen Z, Liu Z, Sun Z, Shao L, Zhao Z, Zhou L (2022) Microbial diversity and biogeochemical cycling potential in deep-sea sediments associated with seamount, trench, and cold seep ecosystems. Front Microbiol 13:1029564

20. Su H, Wu C, Han P, Liu Z, Liang M, Zhang Z, Wang Z, Guo G, He X, Pang J, Wang C, Weng S, He J (2022) The microbiome and its association with antibiotic resistance genes in the hadal biosphere at the Yap Trench. J Hazard Mater 439:129543

21. Zhang X, Xu W, Liu Y, Cai M, Luo Z, Li M (2018) Metagenomics Reveals Microbial Diversity and Metabolic Potentials of Seawater and Surface Sediment From a Hadal Biosphere at the Yap Trench. Front Microbiol 9:2402

Table S2. Environmental and geochemical factors throughout the sediment core

| Sampling depth (cmbsf; DNA extraction) | Median particle size (μm) | TOC  (%) | TN (%) | TOC/TN (mol/mol) | δ^13^C (‰) | TOC-^14^C age (yr) | PO_4_^3-^  (μmol/L) | NH_4_^+^  (μmol/L) | SiO_3_^2-^  (μmol/L) | NO_2_^-^  (μmol/L) | NO_3_^-^  (μmol/L) | SO_4_^2-^  (mmol/L) |
| --- | --- | --- | --- | --- | --- | --- | --- | --- | --- | --- | --- | --- |
| 0-1 | 13.7 | 0.42 | 0.081 | 6.03 | -20.93 | 5090±95 | 4.23 | 75.51 | 319.50 | 0.28 | 23.92 | 25.39 |
| 1-2 | 14 | 0.47 | 0.072 | 7.58 | -21.73 | 5865±100 | 3.65 | 74.49 | 356.35 | 0.21 | 22.08 | 24.34 |
| 2-4 | 15.7 | 0.42 | 0.080 | 6.07 | -20.62 | 4970±95 | 3.15 | 80.89 | 356.80 | 0.66 | 24.39 | 25.96 |
| 4-6 | 13.7 | 0.46 | 0.070 | 7.68 | -21.58 | 5580±95 | 3.00 | 74.43 | 327.68 | 0.32 | 18.61 | 24.37 |
| 6-8 | 20 | 0.46 | 0.070 | 7.58 | -21.51 | 5630±95 | 3.73 | 82.95 | 380.40 | 0.38 | 20.66 | 25.14 |
| 8-10 | 15.2 | 0.47 | 0.070 | 7.87 | -21.74 | 5915±95 | 3.66 | 72.86 | 331.19 | 0.39 | 19.41 | 24.31 |
| 10-12 | 14.9 | 0.46 | 0.083 | 6.44 | -21.35 | 5270±90 | 3.23 | 73.03 | 334.85 | 0.40 | 22.44 | 25.76 |
| 12-15 | 9.24 | 0.49 | 0.071 | 8.08 | -21.61 | 5295±95 | 0.74 | 40.94 | 281.52 | 0.33 | 25.84 | 24.87 |
| 15-18 | 11.1 | 0.44 | 0.068 | 7.50 | -21.59 | 5195±95 | / | 18.49 | 253.42 | 0.38 | 25.86 | 23.16 |
| 18-21 | 16.3 | 0.46 | 0.066 | 8.10 | -21.49 | 5110±95 | / | 9.84 | 268.68 | 0.36 | 24.03 | 23.19 |
| 21-24 | 14.6 | 0.43 | 0.063 | 7.98 | -21.53 | 5370±95 | 0.22 | 5.95 | 309.55 | 0.24 | 15.80 | 25.68 |
| 24-27 | 9.17 | 0.45 | 0.066 | 7.92 | -21.28 | 5130±95 | / | 7.37 | 284.45 | 0.56 | 12.41 | 25.71 |
| 27-28 | 10.9 | 0.50 | 0.065 | 8.99 | -21.70 | 5815±95 | / | 6.56 | 326.52 | 0.24 | 7.97 | 26.57 |
| 28-31 | 24.7 | 0.41 | 0.071 | 6.82 | -20.75 | / | 1.05 | 11.67 | 321.59 | 0.19 | 8.36 | 26.44 |
| 31-34 | / | / | / | / | / | / | / | / | / | / | / | / |
| 34-37 | / | / | / | / | / | / | / | / | / | / | / | / |
| 37-40 | 14.1 | 0.44 | 0.085 | 6.08 | -20.70 | 3765±95 | 0.80 | 19.61 | 325.12 | 0.16 | 7.18 | 27.01 |
| 40-43 | / | / | / | / | / | / | / | / | / | / | / | / |
| 43-46 | / | / | / | / | / | / | / | / | / | / | / | / |
| 46-49 | / | / | / | / | / | / | 0.28 | 31.02 | 335.02 | 0.27 | 4.25 | 26.37 |
| 49-52 | 7.35 | 0.42 | 0.074 | 6.60 | -20.04 | / | / | / | / | / | / | / |
| 52-55 | / | / | / | / | / | / | / | / | / | / | / | / |
| 55-58 | / | / | / | / | / | / | / | / | / | / | / | / |
| 58-61 | 11.6 | 0.43 | 0.073 | 6.82 | -20.54 | 4075±110 | 0.88 | 39.67 | 335.50 | 0.24 | 4.66 | 26.67 |
| 61-64 | / | / | / | / | / | / | / | / | / | / | / | / |
| 64-67 | / | / | / | / | / | / | / | / | / | / | / | / |
| 67-70 | / | / | / | / | / | / | 0.99 | 47.78 | 361.61 | 0.28 | 5.26 | 26.73 |
| 70-73 | / | / | / | / | / | / | / | / | / | / | / | / |
| 73-76 | / | / | / | / | / | / | / | / | / | / | / | / |
| 76-78 | / | / | / | / | / | / | / | / | / | / | / | / |
| 78-81 | 9.43 | 0.43 | 0.076 | 6.55 | -20.59 | / | 1.49 | 60.04 | 451.02 | 0.20 | 7.66 | 25.47 |
| 81-84 | / | / | / | / | / | / | / | / | / | / | / | / |
| 84-87 | / | / | / | / | / | / | / | / | / | / | / | / |
| 87-90 | / | / | / | / | / | / | / | / | / | / | / | / |
| 90-93 | 9.9 | 0.43 | 0.076 | 6.65 | -20.30 | 4265±110 | 1.02 | 66.40 | 314.71 | 0.15 | 5.34 | 26.77 |
| 93-96 | / | / | / | / | / | / | / | / | / | / | / | / |
| 96-99 | / | / | / | / | / | / | / | / | / | / | / | / |
| 99-102 | 8.89 | 0.39 | 0.071 | 6.43 | -20.06 | / | 1.31 | 71.80 | 331.87 | 0.09 | 5.29 | 26.46 |
| 102-105 | / | / | / | / | / | / | / | / | / | / | / | / |
| 105-108 | / | / | / | / | / | / | / | / | / | / | / | / |
| 108-111 | 14.9 | 0.36 | 0.066 | 6.38 | -20.21 | 4295±100 | 1.09 | 79.34 | 347.49 | 0.14 | 4.34 | 26.80 |
| 111-114 | / | / | / | / | / | / | / | / | / | / | / | / |
| 114-117 | / | / | / | / | / | / | / | / | / | / | / | / |
| 117-120 | / | / | / | / | / | / | / | / | / | / | / | / |
| 120-123 | 25.4 | 0.37 | 0.067 | 6.47 | -20.33 | / | 2.86 | 89.72 | 391.99 | 0.16 | 4.30 | 27.51 |
| 123-126 | / | / | / | / | / | / | / | / | / | / | / | / |
| 126-129 | / | / | / | / | / | / | / | / | / | / | / | / |
| 129-131 | / | / | / | / | / | / | 1.32 | 97.93 | 404.09 | 0.15 | 5.96 | 27.41 |
| 131-134 | / | / | / | / | / | / | / | / | / | / | / | / |
| 134-137 | / | / | / | / | / | / | / | / | / | / | / | / |
| 137-140 | / | / | / | / | / | / | / | / | / | / | / | / |
| 140-143 | 7.65 | 0.39 | 0.070 | 6.52 | -20.28 | 4365±95 | 2.00 | 103.21 | 378.69 | 0.38 | 24.50 | 25.56 |
| 143-146 | / | / | / | / | / | / | / | / | / | / | / | / |
| 146-149 | / | / | / | / | / | / | / | / | / | / | / | / |
| 149-152 | 6.83 | 0.41 | 0.071 | 6.74 | -20.48 | / | 0.79 | 105.25 | 404.42 | 0.13 | 4.41 | 26.59 |
| 152-155 | / | / | / | / | / | / | / | / | / | / | / | / |
| 155-158 | / | / | / | / | / | / | / | / | / | / | / | / |
| 158-161 | 7.32 | 0.39 | 0.070 | 6.57 | -19.94 | 4595±90 | 1.63 | 110.11 | 395.21 | 0.17 | 7.94 | 26.47 |
| 161-164 | / | / | / | / | / | / | / | / | / | / | / | / |
| 164-167 | / | / | / | / | / | / | / | / | / | / | / | / |
| 167-170 | / | / | / | / | / | / | / | / | / | / | / | / |
| 170-173 | / | / | / | / | / | / | 2.45 | 119.99 | 434.14 | 0.22 | 3.79 | 27.18 |
| 173-176 | / | / | / | / | / | / | / | / | / | / | / | / |
| 176-178 | / | / | / | / | / | / | / | / | / | / | / | / |
| 200-203 | 9.17 | 0.36 | 0.061 | 6.99 | -20.56 | / | 3.56 | 131.47 | 476.24 | 0.25 | 4.31 | 26.41 |
| 225-228 | 12.7 | 0.34 | 0.063 | 6.33 | -20.16 | 4800±115 | 3.38 | 129.40 | 525.89 | 0.10 | 5.86 | 23.52 |
| 250-253 | / | / | / | / | / | / | 3.69 | 135.01 | 417.63 | 0.16 | 4.29 | 26.02 |
| 275-278 | 7.28 | 0.40 | 0.069 | 6.83 | -20.28 | 4630±100 | 2.71 | 135.66 | 392.16 | 0.09 | 5.39 | 25.92 |
| 300-303 | / | / | / | / | / | / | 2.73 | 144.12 | 399.69 | 0.11 | 3.56 | 26.63 |
| 328-331 | 10.5 | 0.40 | 0.069 | 6.68 | -20.29 | 4520±95 | 1.82 | 144.99 | 383.35 | 0.16 | 4.09 | 26.43 |
| 354-357 | / | / | / | / | / | / | 2.10 | 152.04 | 340.43 | 0.08 | 3.09 | 26.28 |
| 381-384 | 8.43 | 0.37 | 0.063 | 6.76 | -20.53 | 4395±100 | 2.20 | 143.31 | 392.70 | 0.09 | 4.68 | 24.38 |
| 407-410 | / | / | / | / | / | / | 1.07 | 152.82 | 378.25 | 0.09 | 3.15 | 26.32 |
| 434-437 | 8.33 | 0.36 | 0.063 | 6.69 | -20.29 | 4920±95 | 0.08 | 152.24 | 389.52 | 0.16 | 4.23 | 26.74 |
| 460-463 | / | / | / | / | / | / | 0.77 | 158.06 | 373.97 | 0.06 | 2.82 | 26.90 |
| 487-490 | 10.3 | 0.35 | 0.062 | 6.62 | -20.25 | 5150±95 | 1.09 | 147.14 | 354.38 | 0.11 | 4.13 | 25.84 |
| 539-542 | 11.4 | 0.33 | 0.058 | 6.61 | -19.84 | / | 0.74 | 161.56 | 396.37 | 0.08 | 4.95 | 26.57 |
| 591-594 | 13.7 | 0.40 | 0.066 | 7.07 | -19.90 | / | 7.64 | 158.61 | 434.81 | 0.10 | 4.28 | 25.17 |
| 644-647 | 10.4 | 0.27 | 0.049 | 6.43 | -19.89 | / | 9.94 | 161.77 | 467.39 | 0.08 | 6.07 | 25.63 |
| 697-700 | 17 | 0.32 | 0.056 | 6.70 | -19.97 | / | 7.00 | 151.13 | 482.40 | 0.09 | 8.64 | 22.53 |
| 749-752 | 15.2 | 0.32 | 0.055 | 6.73 | -20.03 | / | 7.11 | 185.70 | 461.61 | 0.13 | 18.87 | 24.49 |

Table S3. Enzymes and substrates used in the present study to measure extracellular enzyme activity in the Challenger Deep sediments.

| Substrate | Enzymes | Characterization | Function |
| --- | --- | --- | --- |
| 4-MUF-*β*-D-glucopyranoside | *α*-glucosidase | glycosidase | Carbon-acquiring enzyme |
| 4-MUF-*β*-D-glucopyranoside | *β*-glucosidase | glycosidase | Carbon-acquiring enzyme |
| 4-MUF-*β*-D-xylopyranoside | *β*-xylosidase | glycosidase | Carbon-acquiring enzyme |
| 4-MUB-*β*-D-cellobioside | Cellobiase | glycosidase | Carbon-acquiring enzyme |
| 4-MUF-N-acetyl*-β*-D-glucosaminide | N-acetyl-*β*-D-glucosaminidase | glycosidase | Carbon-acquiring enzyme |
| L-Leucine-4-AMC*HCl | Aminopeptidase | exopeptidase | Nitrogen-acquiring enzyme |
| 4-MUF-butyrate | Lipase | esterase | - |
| 4-MUF-phosphate | Phosphatase | esterase | Phosphorus-acquiring enzyme |
| 4-MUF-sulfate potassium salt | Sulfatase | esterase | - |
